# The DNA methylation landscape of multiple myeloma shows extensive inter- and intrapatient heterogeneity that fuels transcriptomic variability

**DOI:** 10.1186/s13073-021-00938-3

**Published:** 2021-08-09

**Authors:** Jennifer Derrien, Catherine Guérin-Charbonnel, Victor Gaborit, Loïc Campion, Magali Devic, Elise Douillard, Nathalie Roi, Hervé Avet-Loiseau, Olivier Decaux, Thierry Facon, Jan-Philipp Mallm, Roland Eils, Nikhil C. Munshi, Philippe Moreau, Carl Herrmann, Florence Magrangeas, Stéphane Minvielle

**Affiliations:** 1 Université de Nantes, CNRS, INSERM, CRCINA, Nantes, F-44000 France; 2 Institut de Cancérologie de l’Ouest, Nantes-Saint Herblain, France; 3 LS2N, CNRS, Université de Nantes, Nantes, France; 4 Centre Hospitalier Universitaire, Nantes, France; 5 Institut Universitaire du Cancer, CHU, Centre de Recherche en Cancérologie de Toulouse, INSERM 1037, Toulouse, France; 6 Centre Hospitalier Universitaire, Rennes, France; 7 Centre Hospitalier Universitaire, Lilles, France; 8 Research Group Genome Organization & Function, DKFZ, and BioQuant Heidelberg, Heidelberg, 69120 Germany; 9 Health Data Science Unit, Medical Faculty Heidelberg and BioQuant, Heidelberg, 69120 Germany; 10 Universitätsmedizin Berlin, corporate member of Freie Universität Berlin, Humboldt- Universität zu Berlin, and Berlin Institute of Health, Charitéplatz 1, Berlin, 10117 Germany; 11 Berlin Institute of Health (BIH), Center for Digital Health, Anna-Louisa-Karsch-Strasse 2, Berlin, 10178 Germany; 12 Dana-Farber Cancer Institute, Harvard Medical School, LeBow Institute for Myeloma Therapeutics and Jerome Lipper Center for Multiple Myeloma Research, Boston, MA United States

**Keywords:** Multiple myeloma, Disordered DNA methylation, Epipolymorphism, Epiallele switching, Inter- and intrapatient heterogeneity, Transcriptomic variability

## Abstract

**Background:**

Cancer evolution depends on epigenetic and genetic diversity. Historically, in multiple myeloma (MM), subclonal diversity and tumor evolution have been investigated mostly from a genetic perspective.

**Methods:**

Here, we performed an analysis of 42 MM samples from 21 patients by using enhanced reduced representation bisulfite sequencing (eRRBS). We combined several metrics of epigenetic heterogeneity to analyze DNA methylation heterogeneity in MM patients.

**Results:**

We show that MM is characterized by the continuous accumulation of stochastic methylation at the promoters of development-related genes. High combinatorial entropy change is associated with poor outcomes in our pilot study and depends predominantly on partially methylated domains (PMDs). These PMDs, which represent the major source of inter- and intrapatient DNA methylation heterogeneity in MM, are linked to other key epigenetic aberrations, such as CpG island (CGI)/transcription start site (TSS) hypermethylation and H3K27me3 redistribution as well as 3D organization alterations. In addition, transcriptome analysis revealed that intratumor methylation heterogeneity was associated with low-level expression and high variability.

**Conclusions:**

We propose that disrupted DNA methylation in MM is responsible for high epigenetic and transcriptomic instability allowing tumor cells to adapt to environmental changes by tapping into a pool of evolutionary trajectories.

**Supplementary Information:**

The online version contains supplementary material available at (10.1186/s13073-021-00938-3).

## Background

Multiple myeloma (MM) is a neoplasm of plasma cells (PCs) with an incidence rate of approximately 5/100,000 in Europe. The median survival time of patients has improved substantially over the past decade. This is due to the establishment of high-dose therapy followed by autologous stem cell transplantation as a routine procedure, significant improvements in supportive care strategies, and the introduction and widespread use of drugs including immunomodulatory drugs, proteasome inhibitors, histone deacetylase inhibitors, and monoclonal antibodies. Nevertheless, almost all patients ultimately relapse due to the emergence of more aggressive subpopulations of myeloma PCs resistant to therapeutic agents.

Several mechanisms have been suggested to explain the capacity of the subpopulations of myeloma cells within an individual to survive the pressure of frontline therapy and proliferate. These mechanisms include the emergence of myeloma cells that achieve bortezomib resistance by decommitment from immunoglobulin synthesis [1] or by the derepression of growth factor receptors typically not associated with the plasma cell lineage [2], somatic mutations that emerge during disease progression involving key driver genes in MM such as the mono- or biallelic loss of *TP53* [3, 4] or the biallelic loss of *TRAF3* [5]. These mechanisms facilitate the expansion of proliferative subclonal populations. Genetic intratumor heterogeneity increases the evolutionary fitness potential of a rare subset of myeloma cells harboring a particular combination of molecular aberrations to survive when challenged by multiagent chemotherapy [4]. The disease evolves predominantly through a Darwinian process of clonal expansion, and the population of tumor PCs represents an admixture of competing genetic subclones [5–8]. In most patients, the treatment pressure causes the profound reorganization and diversification of subclonal populations with complex dynamics of tumor evolution raising the possibility of biologically and clinically important cross-talk between subclones [9]. However, the small number of genetic alterations detected in MM and relevant in relapse mechanisms does not alone explain the profound phenotypic variability across patients [4, 10]. Beyond genetic diversity, other processes generate the intratumoral functional heterogeneity of cancer cells, including global epigenetic changes [11].

Among epigenetic alterations, aberrant DNA methylation is a common trait of cancer cells characterized by global hypomethylation with focal hypermethylation of CpG islands (CGIs) promoters [12, 13]. Hypomethylation is associated with genomic instability [14] whereas locally hypermethylation likely contributes to the silencing of tumor suppressor genes [15, 16]. Previous studies have shown that genome wide hypomethylation is also accompanied by a global increase of entropy in most cancers [17, 18]. Furthermore, increased intrasample methylation heterogeneity in cancers compared to their normal counterparts is associated with more pejorative outcomes in hematological malignancies [19–21]. So far, no such study has been carried out in MM. To explore the disrupted DNA methylation landscape in MM, we conducted an analysis of 42 myeloma samples from 21 patients by using enhanced reduced representation bisulfite sequencing (eRRBS). We found that the aberrant DNA methylation is associated with a high intratumor heterogeneity and that combinatorial entropy changes between normal and malignant plasma cells are significantly linked to survival outcome in this pilot study. Moreover, we showed the importance of the DNA methylation disruption on inter- and intrapatient heterogeneity and transcriptomic variability.

Collectively, our data highlight the clinical and functional roles of epigenetic heterogeneity during MM development.

## Methods

### Cohorts and samples

Forty-two PC samples derived from 21 MM patients were employed for eRRBS analysis. These bone marrow samples were collected at different stages of the disease: 17 diagnosis (MMD)/relapse (MMR) paired samples, 3 smoldering multiple myeloma (SMM)/MMD paired samples, and 1 SMM/MMR paired sample. Control PCs were purified from bone marrow samples lacking abnormal plasma cells from 3 patients suspected of monoclonal gammopathy of 1 undetermined significance (MGUS). Patients, (11 women and 10 men, with a median age of 59 years) and control patients (1 woman and 2 men, with a median age of 52 years) were monitored at the Intergroupe Francophone du Myélome (IFM) centers, and all provided informed consent. These samples were analyzed using different metrics in order to determine intratumor heterogeneity (epipolymorphism and PDR) and interstates heterogeneity (combinatorial entropy, eloci, EPM) (Additional file 1: Table S1). RNA-seq analysis was also performed for these 42 PC samples.

PC samples derived from 10 MMD patients were employed for WGBS analysis among them 5 were in common with eRRBS analysis. In addition, we used publically available datasets from BLUEPRINT Consortium [22] including 3 MMD patients and 2 control patients. All these samples were used to PMDs identification through R package MethylSeekR [23]. Chip-seq of histone marks (H3K9me3 and H3K27me3) from the 3 MMD patients (BLUEPRINT Consortium [22]) was used to analyze the redistribution of repressive histone marks in PC-PMDs.

### Sample preparation and nucleic acid purification

Myeloma cells were purified using nanobeads and an anti-CD138 antibody (RoboSep, Stem Cell Technologies). After immunomagnetic sorting, the purity of the plasma cell suspension was verified, and only samples with at least 85% of PCs were subjected to genomic analysis. The average cell purity of MM was > 99% (range 90–100%). Control PCs were purified using the same procedure as for myeloma cells. The average purity of control PCs was 81% (range 74–86%). The absence of abnormal plasma cells in the bone marrow aspiration was assessed by flow cytometry using the 7-color combination CD45, CD19, CD38, CD138, CD28+CD56, kappa, and lambda chains published previously [24]. The DNA and RNA of CD138+ cells were purified using the Qiagen protocol and the quality and quantity of the nucleic acids were measured on the Nanodrop, Qubit, and Agilent profiles and stored in the MM biobanks of the Nantes and Toulouse Hospitals.

### Chromosomal abnormalities analysis

Chromosomal abnormalities present in tumor plasma cells were detected either by fluorescence in situ (FISH) for t(4;14)(p16;q32) and del(17p) using specific provided by Abbott Molecular (Paris, France) and Cytocell (Paris, France) according to the manufacturer’s instructions. The del(17p) status was retained only if present in at least 55% of the plasma cells or by single nucleotide polymorphism (SNP) array for 1q gain, del(1p32) targeting CDKN2C and hyperdiploidy previously defined as 47 chromosomes or more [25]. Hybridization-based genomic profiling arrays were performed using Genome-Wide Human SNP Array 6.0 or the CytoScan HD array, according to the manufacturer’s instructions (Affymetrix, Santa Clara, CA, USA) now part of Thermo Fisher Scientific (Thermo Fisher Scientific, Inc.). Following the procedures of sample preparation, hybridization, and scanning, the CEL file of Genome-Wide Human SNP Array 6.0 was analyzed as previously described [5] and the CEL file of CytoScan HD array was analyzed using the Chromosome Analysis Suite (ChAS) software (Thermo Fisher Scientific, Inc). Chromosomal changes of each sample were visualized by a diagram of all CNVs, the karyoview, and by visual inspection. Ploidy of tumor plasma cells for each patient was obtained using allele-specific copy number analysis of tumors algorithm (ASCAT 2.2) with default parameters [26].

### eRRBS

eRRBS is an improvement of the reduced representation bisulfite sequencing (RRBS) protocol, resulting in an increase in CpG detection and coverage. eRRBS library preparations were performed by Integragen and adapted from the protocol described by Garrett-Bakelman et al. [27]. DNA was digested with the Msp1 enzyme, fragments between 150 bp and 400 bp were selected, and bisulfite conversions were processed. Libraries were sequenced on a HiSeqTM 4000 Illumina machine using 75 bp paired-end reads to an average depth of 50X per covered CpG. The average number of reads sequenced per patient was 55,216,285, and the average alignment rate of uniquely mapped reads was 60.81%, with an average of 2,712,252 CpGs per patients with a coverage of 10X and an average of 1,538,510 CpGs per patient with a coverage of 60X (Additional file 2: Table S2).

### eRRBS analysis

The adaptor sequences were removed by Cutadapt (version 1.10) [28]. FastQC (version 0.11.4) was used for quality control of the Illumina paired-end sequencing data [29]. Bisulfite reads were aligned to the bisulfite-converted hg19 genome with the nondirectional model of Bismark alignment software (version 0.14.1) [30]. CpG methylation levels were obtained using the R package methylKit with the default settings: a minimum of 10 reads covering a CpG and at least 20 PHRED quality scores by CpG [31]. We calculated the epigenetic changes between two stages using methclone [32], an algorithm that detects loci of 4 adjacent CpGs (minimum depth of 60 reads), called epialleles (16 possible patterns according to CpG methylation).

### Metrics used to calculate DNA methylation heterogeneity

Epipolymorphism (Epi) was calculated for each locus of the four adjacent CpGs covered by the same read, to measure intratumor epigenetic heterogeneity, as previously described by Landan et al. [33]:

$Epi=1-\sum _{i=1}^{16}p_{i}^{2}$ where *p*_*i*_ is the proportion of the epiallele i. The minimal epipolymorphism is 0 (only one pattern represented); epipolymorphism cannot have a value greater than 1. The maximum value of epipolymorphism is 0.9375 (when all 16 patterns are equally distributed).

To quantify the degree of epiallele pattern shift, methclone was used to compute the entropy difference (*Δ*S) and thus compare the distributions of epialleles between different stages. The entropy difference value can range from 0 (no change) to – 144 (maximum change). Loci are characterized as eloci when *Δ*S < - 70 (corresponding to a significant entropy shift). The methclone algorithm allows the discovery, quantification and ranking of subclonal selection based on epiallele shifts. This allows us to measure clonal evolution between disease stages and epigenetic heterogeneity.

To normalize and compare the number of eloci per patient, we computed the number of eloci per million loci sequenced (EPM) as previously described by Sheng Li et al. [32]:

$EPM=\frac {10^{6}}{C}\times E$where E is the total number of eloci detected between the two stages and C is the total number of loci covered by both samples.

A locus between the NPC and diagnosis samples was defined as an elocus only if it was annotated as an elocus with the 3 NPCs. A hypermethylated or hypomethylated elocus was defined as a DNA methylation difference of at least 25%.

The PDR variable was defined as the percentage of epialleles exhibiting heterogeneous DNA methylation (i.e., not fully methylated or unmethylated).

Four possible epiallele pattern changes between two stages were determined as follows: disorder maintenance: no predominant pattern (all patterns below 30%) at either stages; selection: no predominant pattern at stage 1 (all patterns below 30%), one pattern becomes highly predominant (above 70%) at stage 2; disorder: one pattern is highly predominant (above 70%) at stage 1, no predominant pattern (all patterns below 30%) at stage 2; switch: both stages have a highly predominant pattern (above 70%) but patterns of both stages are different.

### WGBS

Data quality control and adaptor trimmed were performed with the Trimmomatic tool [34]. Read mapping was carried out with the methylCtools aligner [35]. methylCtools uses an alignment approach similar to that of Bismark by improving the handling of large amounts of data and the speed of alignment. CpG extraction and methylation analyses were carried out with methylCtools and the bsseq R package [36], allowing the analysis, management, and storage of WGBS data. An average depth of 17.2X per covered CpG was observed. As with eRRBS data, we set a coverage threshold of a minimum of 10X for each CpG sequenced for our analyses, approximately 13.4 million of CpGs per sample with a coverage of 10X were observed. The dmrseq R package was used to visualize WGBS data and smooth methylation signals [37].

### Detecting PMDs

PMDs were detected in WGBS samples using the R package MethylSeekR [23]. Prior to the detection of PMDs, CpGs overlapping with common single-nucleotide polymorphism (SNPs) were removed from the data (dbSNP137commonhg19, version 1.0.0). The distribution of *α* values was used to determine the presence or absence of PMDs in samples. When the *α* distribution was bimodal, the segmentPMDs function was run to identify PMDs on the genome by a hidden Markov model.

### RNA sequencing (RNA-seq)

RNA-seq was performed using 200 ng of total RNA by GATC Biotech. Directional libraries were generated after mRNA selection by polyA selection using the UTP method. RNA-seq libraries were sequenced on a HiSeq 2500 Illumina machine using 100 bp paired-end reads. The average number of reads sequenced per sample was 68 454 433. The average alignment rate of uniquely mapped reads was 66% (corresponding to 48,034,921 reads). Read alignment was performed using the STAR aligner (version 2.4.0f1) [38] and human genome hg19 as the reference. FastQC (version 0.11.4) [29] was used for basic quality control of the Illumina paired-end sequencing data. PCR duplicates were determined and removed using the Picard algorithm [39]. The number of reads mapped to each gene was calculated using HTSeq-count, part of the HTSeq framework [40], version 0.6.0. We then normalized the mapped read counts per million of mapped fragments (FPM) using the robust median ratio method with the DESeq2 R package [41].

### Hi-C data analysis and compartments A/B

Hi-C datasets were downloaded for three cell lines: GM12878 (GSM1608505), RPMI-8226 (GSM2334832), and U266 (GSM2334834). HiC-Pro software (version 2.10.0) [42] was used to process Hi-C data, from raw-data to normalized contact maps. All reads were mapped to hg19 using Bowtie2 (global parameters: –very-sensitive -L 30 –score-min L,-0.6,-0.2 –end-to-end –reorder; local parameters:–very-sensitive -L 20 –score-min L,-0.6,-0.2 –end-to-end –reorder). Contact maps were generated at 20 kb resolution and normalized by the iterative correction and eigenvector decomposition (ICED) technique.

The 20-kb resolution intrachromosomal contact matrices generated by HiC-Pro were used as input to determine compartments A/B and to annotate and visualize interaction maps with R package HiTC version 1.22.1 [43]. Principal component analysis was used to separate chromatin into two compartments: compartment A, with higher gene density, and compartment B, with lower gene density. The determination of compartments A and B was estimated by the analysis of the eigenvectors of the genome contact matrix by the observed-expected method. On the basis that changes in the sign of the eigenvector of the contact matrix correspond to the limits of the genome compartments and taking into account gene density, the compartmentalization of the genome was defined.

### TADs

Hi-C data were used to determine the TADs with the HiCExplorer tool, a set of programs used to process, normalize, analyze, and visualize Hi-C data [44]. TADs were defined with hicFindTADs, and hicPlotTADs was used to visualize the TADs. For the comparison of PMD and TAD borders, we generated a set of randomized PMDs with a size and genomic distributions similar to those of real PMDs. We then calculated the distance between TAD borders and, on the one hand, PMD borders, and, on the other hand, random PMD borders. A t test was carried out to compare the distributions of these distances.

### Genomic annotation

RefSeq annotation and CpG islands were obtained from UCSC (https:/garance/genome.ucsc.edu/ ) using the February 2009 (GRCh37/hg19) assembly. CpG shores were defined as regions flanking 2 kb of CpG islands, and CpG shelves were defined as regions flanking 2 kb of CpGs shores.

HOMER was used to annotate loci and eloci, using the annotatePeaks.pl script [45], which determines the genomic type annotation occupied by the center of the loci. We used their basic annotation, including TSSs, transcription termination sites (TTSs), exons, introns, and intergenic regions. We defined the promoters as regions flanking 2 kb of the TSS, and we defined promoter CGIs as promoters that intersect with CpG islands.

All 127 reference epigenomes with 25 chromatin state segmentation annotations were downloaded from the NIH Roadmap Epigenomics Project (https://egg2.wustl.edu/roadmap/data/byFileType/chromhmmSegmentations/ChmmModels/imputed12marks/jointModel/final/;all\OT1\25_imputed12marks_mnemonics.bed.gz\OT1\files).

To measure the intersections between regions, bedtools was used (more precisely, the “intersect” option) [46].

Data from the BLUEPRINT Consortium were converted to hg19 coordinates using the liftOver tool [22, 47].

### Functional annotation

GREAT tools (version 3.0.0) was used to assign biological functions by analyzing the annotations of the nearby genes [48]. Enrichment statistics were computed using the binomial test and the hypergeometric gene-based test. Pathways were selected as significantly enriched if the false discovery rate (FDR q value) was < 0.01.

The Database for Annotation, Visualization and Integrated Discovery (DAVID) web interface (version 6.7) was used to perform functional enrichment analysis from a list of genes, especially the KEGG pathways functional database [49]. Enrichment statistics were computed using the Fisher test. For the significance threshold, we have considered genes as greatly enriched if the annotation categories yielded a *p* value less than 0.1 (DAVID default threshold)[50].

### Survival analysis

Time to relapse data were available for 17 patients. For the relapse-free survival (RFS) analysis, the survival endpoint in this study was the time from diagnosis until relapse. The patients were divided into two groups by the median EPM value: low and high EPMs. Survival curves were estimated using the Kaplan-Meier method and compared with the log-rank test.

For all statistical tests in this study, a two-sided *p* value of 0.05 was considered statistically significant. All statistical analyses were performed with software R 3.5.1 [51], in addition to the packages already mentioned in the text, packages data.table [52], viridis, ggbio, GenomicRanges, RColorBrewer, biovizBase, grid, gridBase, MASS, fields, KernSmooth, sp, org.Hs.eg.db, DBI, and survival were used.

## Results

### Aberrant DNA methylation landscape is associated with an intratumor heterogeneity in MM

Before exploring intratumor DNA methylation heterogeneity, we confirmed using WGBS data that MM is a very hypomethylated tumor with focal hypermethylation (Additional file 1: Table S1; Additional file 3: Figure S1,S2a), as previously reported [8, 53]. Individual CpG sites showed a predominantly bimodal pattern in MM patients (Additional file 3: Figure S2b) with methylation levels depending on the local density of CpG (Additional file 3: Figure S2c; Additional file 4: Table S3). In contrast, control bone marrow plasma cells displayed striking differences in their global methylation status with a significant level of partially methylated regions that progressively increased during the terminal differentiation of germinal center B cells into tonsillar plasma cells and long-lived bone marrow plasma cells (Additional file 3: Figure S3; BLUEPRINT consortium [53]). This is likely to be due to the multiple rounds of mitotic division coupled with DNA demethylation that plasmablasts and plasma cells undergo during differentiation [54, 55]. Hence, an interesting feature of MM is that the global hypomethylation is apparently reactivated as malignant plasma cells actively divide driving a more bimodal DNA methylation pattern. Although MM displayed no evidence of aberrant DNA methylation at many genes (Additional file 3: Figure S4), we observed slight methylation gain at CpG islands (CGIs) and methylation loss at adjacent shores and shelves regions compared to normal plasma cell (NPCs; Additional file 3: Figure S5a), as illustrated in the genomic region of *DOC2B* (Additional file 3: Figure S5b).

**Fig. 1:**
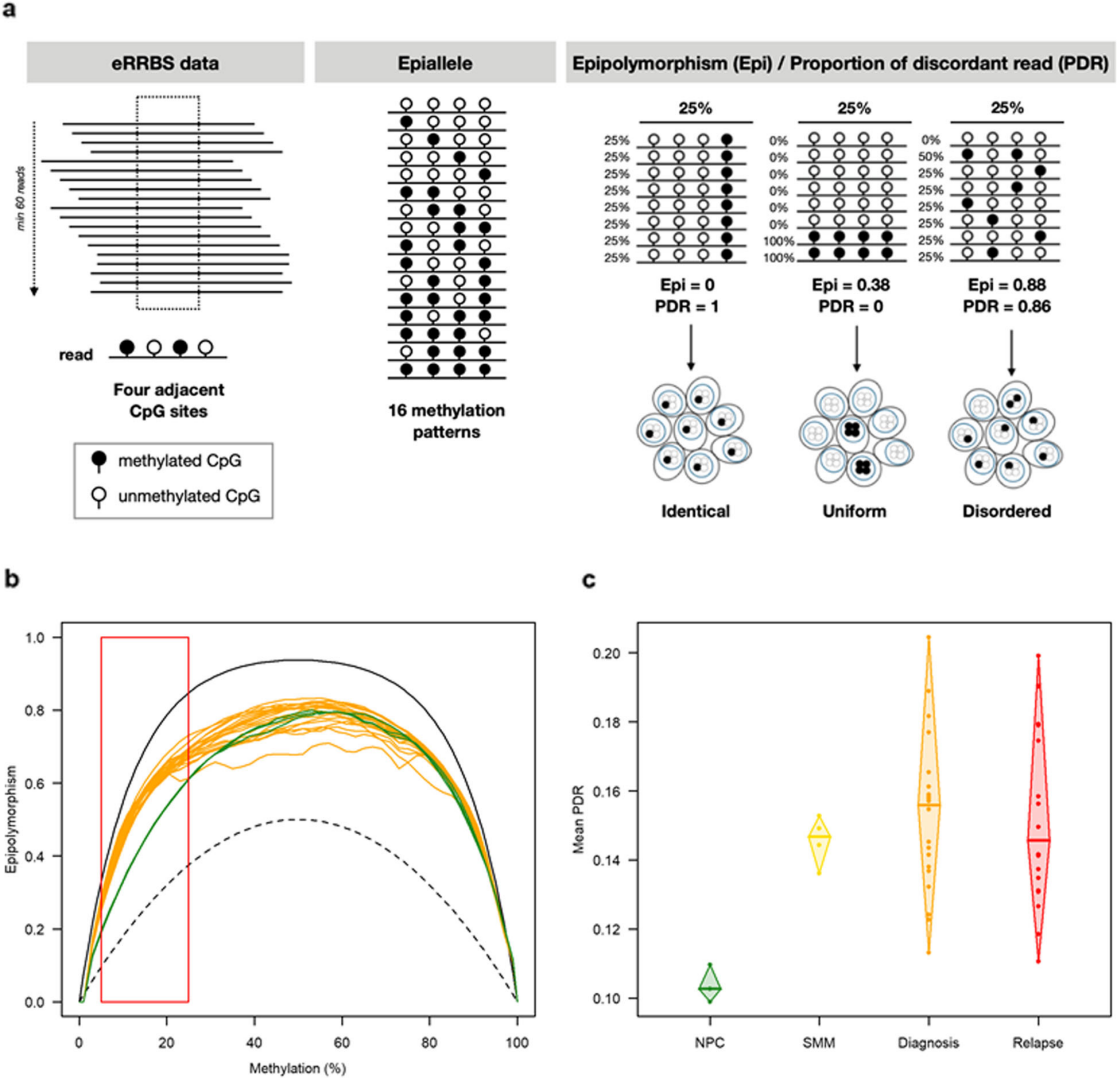
Intrapatient DNA methylation heterogeneity in MM. **a** Locus detection of 4 adjacent CpGs covered by the same read by Methclone tools [32], with at least 60 reads (filled black circle: methylated CpG; empty circle: unmethylated CpG). Sixteen methylation patterns of the epiallele from 4 adjacent CpGs are possible. Assessment of two metrics of epigenetic heterogeneity by locus: the epipolymorphism value (Epi) taking into account the distribution of the probabilities of these 16 possible methylation patterns and the proportion of discordant read value (PDR) taking into account reads with both methylated and unmethylated CpGs. For the same average level of methylation, different Epi and PDR values are possible depending on the bulk. **b** Epipolymorphism levels as a function of the average DNA methylation at each locus in NPC (green) and diagnosis (orange) samples. This color code was used in all figures. The maximal epipolymorphism (continuous black line) and the bimodal epipolymorphism (dotted black line) for each methylation level are represented. **c** Mean proportion of discordant reads per sample

We next explored the degree of DNA methylation intrapatient heterogeneity by using eRRBS technology (Additional file 1: Table S1; Additional file 3: Figure S1). We obtained an average depth of 50X per covered CpG and approximately 2.7 million CpGs per sample, with more than 10X sequencing coverage (Additional file 2: Table S2; Additional file 5: Table S4; Additional file 3: Figure S6). As identical average methylation may correspond to different methylation patterns (identical, uniform, disordered), reflecting homogeneous or heterogeneous cell subpopulation mixture (Fig. 1a), we analyzed eRRBS data applying a computational method that investigates DNA methylation modifications at a genomic locus defined as a group of four contiguous CpGs covered by single sequence reads [32]. We computed for each genomic locus the epipolymorphism, derived from the probability distribution of the 16 possible methylation patterns: a high value of epipolymorphism indicates that, for the specific locus, several of the 16 patterns are present in noteworthy proportions among the different reads, i.e., there is a large inter-read variability, thus a stochastic process of DNA methylation [33]. As expected, almost all genomic loci were located in CGIs (85% in MM samples) and, to a lesser extent, in promoter transcription start sites (TSSs) and gene bodies (74% in MM samples; Additional file 3: Figure S7). We plotted epipolymorphism distribution as a function of methylation level for all loci in NPCs and MM samples (Fig. 1b). Remarkably, loci with modest methylation levels (5–25%) showed a higher degree of epipolymorphism in cancer cells than in control cells whereas at higher methylation levels, epipolymorphism distribution was similar in MM and NPCs, indicating enrichment of a fraction of loci-CGIs with a stochastic methylation state in MM samples. In all cases, promoter CGI methylation gain was associated with an increase in epipolymorphism in MM samples (Additional file 3: Figure S8). This intrasample heterogeneity can come from two origins: a mixture between uniformly methylated reads (i.e., whereby CpGs in an individual read are fully methylated or fully unmethylated, (Fig. 1a, example with mixture of uniformly methylated reads) or variability within reads (i.e., discordant methylation by which CpGs in an individual read are partly methylated; Fig. 1a, example with disordered patterns). In order to better characterize epiallele patterns of each sample, and differentiate between mixture of uniform reads or mixture of disordered patterns, thus assessing the degree of stochasticity in the DNA methylation process, we computed, for each locus, the proportion of discordant reads (PDR), i.e., of reads with both methylated and unmethylated CpGs [19](Fig. 1a). We found that the average PDR was significantly higher (*p* value <0.05, Dunnett-Tukey-Kramer’s pairwise multiple comparison test) in MM samples, regardless of the disease stage, than in NPCs (Fig. 1c). These results demonstrate that methylation heterogeneity in MM arises primarily from variability within DNA reads. We also observed stochastic methylation patterns of MM in loci stratified according to the frequency of fully methylated or fully unmethylated epialleles whereas methylation patterns of NPCs epialleles are almost exclusively uniforms (Additional file 3: Figure S9,S10).

Overall, these results could imply that during disease initiation, growth, and progression, malignant PCs accumulate stochasticity in DNA methylation at the expense of a more coherent methylation state, leading to a high degree of intratumoral epigenetic heterogeneity in MM patients.

### Combinatorial entropy changes are associated with poor survival outcome in our MM cohort

**Fig. 2:**
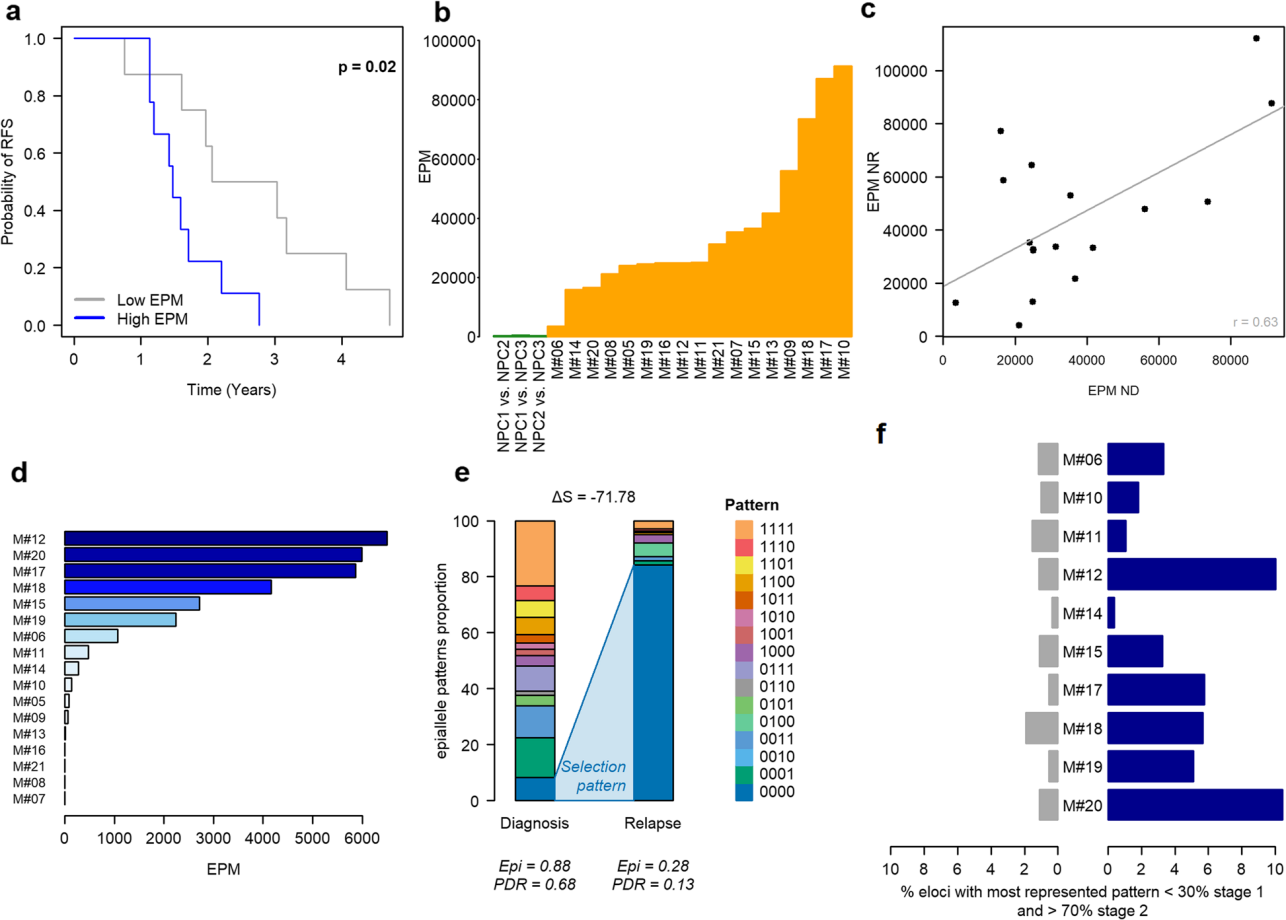
Evolution of epiallelic changes during the progression of MM and their clinical impact. **a** Time to relapse analysis for patients with high (blue, n=9) or low (gray, n = 8) EPM values. **b** EPM values of NPC samples (NPC eloci, in green) and MM samples at diagnosis (MM eloci in orange). **c** Correlation between EPM at diagnosis and relapse compared to that in NPCs. **d** EPM value for each pairwise comparison between diagnosis and relapse (diagnosis vs. relapse eloci). **e** Selection example of a completely unmethylated methylation pattern, minor at diagnosis, but which becomes predominant at relapse for patient M#20, locus position: chr4: 163266538-163266552. **f** Proportion of eloci presenting a selection of a methylation pattern at diagnosis compared to NPCs (in gray) and at relapse compared to diagnosis (in blue)

Given that, in contrast to diffuse large B cell lymphoma, we did not find a clinical relevance linked to the level of intrapatient heterogeneity [21] (Additional file 3: Figure S11), we used another DNA methylation heterogeneity metric which measures shifting between samples rather than variability within individual specimens [32]. The algorithm allows the identification of epigenetic loci (i.e., eloci) that have a significant epiallele composition change (i.e., combinatorial entropy change) between two states (e.g., normal vs. cancer or diagnosis vs. relapse) (Additional file 3: Figure S12). The number of eloci was normalized according to the number of covered loci, referred to as eloci per million loci covered (EPM) to compare genome-wide combinatorial entropy changes across patient samples (Additional file 3: Figure S12)[32] and patients were separated into two groups according to their EPM values. In this pilot study, we found that MM patients with high EPM had a significantly reduced relapse-free survival (*p* value = 0.02 (log-rank test); Fig. 2a). In order to assess whether the prognostic impact of EPM was independent of chromosomal abnormalities, we performed multivariable Cox regression analyses. Five specific chromosomal abnormalities, del(17p), t(4;14), del(1p32), 1q gain, and hyperdiploidy, were tested, as they were recently shown to improve classification of MM patients in the high-risk category for death (Additional file 1: Table S1; Additional file 3: Figure S13a,b)[56]. The results showed that in all regression models tested, EPM remained significant in our cohort (Additional file 6: Table S5) and suggested that combinatorial entropy changes may be indicative of more aggressive disease independently of high-risk genetic lesions. EPM was also independent of age, sex, and ploidy. These results will need further validation in an independent cohort.

Remarkably, the extent of combinatorial entropy changes varied greatly between patients at diagnosis and persisted at relapse compared to NPCs (Fig. 2b, c). To further examine the impact of treatment on combinatorial entropy changes, we assessed the epiallele shifting level during time to relapse by comparing diagnosis vs relapse paired samples (Fig. 2d). The degree of difference was highly variable from one patient to another; 24% of patients showed no epigenetic changes (EPM = 0) while 42% showed substantial changes (EPM > 1000) between diagnosis and relapse. Then, we investigated whether a methylation pattern could be selected in response to treatment similarly to genetic subclones evolution; we compared epiallele shifts between NPCs and diagnosis samples and between paired samples at diagnosis and relapse. Interestingly, among the four possible epiallele pattern changes (Additional file 3: Figure S14), the selection pattern was significantly enriched at relapse compared to diagnosis (*p* value (paired Wilcoxon) = 0.0059, Fig. 2e, f). Although this pattern involved a small number of eloci (median value = 4.2%), this finding suggests that treatment escape is associated with clonal selection at specific genomic loci.

We next sought to investigate the genomic context in which the combinatorial entropy changes occur. To answer this question, we analyzed the distribution of eloci using 127 cell/tissue types at 25-state chromatin state segmentation [57]. We observed an enrichment of eloci in bivalent promoters and quiescent states (Additional file 3: Figure S15a). We obtained similar results by analyzing published ChromHMM data for tonsil plasma cells [58] (Additional file 3: Figure S15b). Bivalent promoters are typically associated with CGIs; as expected, the distribution of eloci in bivalent promoters showed a predominant location at CGIs and promoter-TSS, whereas eloci in quiescent states overlapped with intronic and intergenic regions (Additional file 3: Figure S16a,b).

### Stochastic methylation gains at developmental genes promoters are associated with a decoupling relationship between methylation and gene expression level

**Fig. 3:**
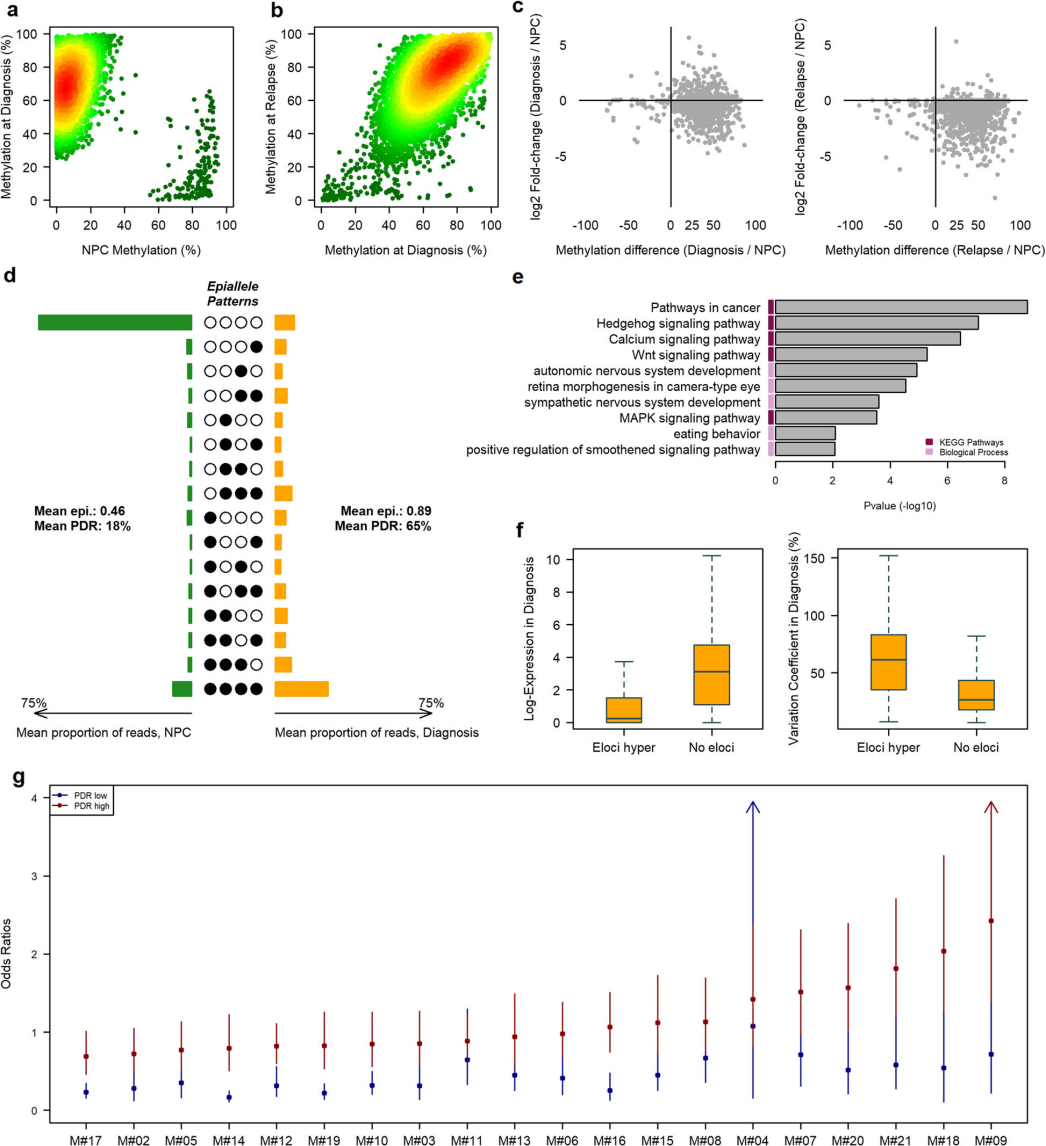
Methylation disruption in bivalent promoters in MM. Scatterplot of eloci in bivalent promoters as a function of DNA methylation in NPC and diagnosis (**a**) and at diagnosis and relapse (**b**) for patient M#17. The color gradient corresponds to the point density (low is green; high is red). **c** Scatterplot of DNA methylation of the eloci promoters versus RNA expression of the associated genes. **d** Average methylation epiallele patterns of eloci in promoter CGIs for NPC and diagnosis samples. **e** Ontological analysis of genes with a bivalent promoter CGI affected by hypermethylated eloci. **f** Normalized expression values of genes with a promoter CGI containing at least one hypermethylated elocus or no elocus (left) and the variation coefficient of these genes in MM samples (right). **g** Odds ratios with 95% confidence intervals for the association between gene expression (FPM >1) and promoter methylation (average methylation > 0.75 / average methylation < 0.25) for genes with high (red) or low (blue) PDR levels in the promoter

Bivalent promoters play an important role during embryonic development. In the embryonic system, bivalent promoter genes are not regulated by DNA methylation but rather by the simultaneous presence of the repressive mark H3K27me3 and the active transcription mark H3K4me3, which allows low basal transcription states that are dynamically inducible to ensure a balance between self-renewal and lineage commitment [59]. Various studies have reported that these promoters are hypermethylated in cancer cell lines and primary tumors [60, 61]. As expected, 82.5% of combinatorial entropy changes found in bivalent promoters were in bivalent promoters of embryonic stem cells (ESCs) and most of the eloci acquired extensive gains of DNA methylation during neoplastic transformation (Fig. 3a and Additional file 3: Figure S17). In addition, DNA methylation mean of promoters with eloci was higher than the mean of promoters without eloci in all patients (Additional file 3: Figure S18). Methylation gains were maintained during progression (Fig. 3b; Additional file 3: Figure S17). These methylation gains were associated with a decrease in gene expression in diagnosis and relapse samples compared to NPCs. Interestingly, upregulated genes were present mostly at the time of diagnosis in almost all patients (Fig. 3c; Additional file 3: Figure S19). These results suggest the existence of a subpopulation which would not have undergone hypermethylation of promoters and which would overexpress certain genes. In this case, a uniform methylation pattern should be predominant. To further characterize these methylation gains, we computed the average epiallele distribution in diagnosis and control samples. Contrary to the expected hypothesis, our results revealed mostly disordered methylation gains at bivalent promoters, reflected by both high PDR and epipolymorphism instead of mixture of uniform states (Fig. 3d).

To investigate the biological relevance of disorder methylation gains, we firstly performed gene ontology (GO) analysis of the genes targeted by hypermethylated eloci. We found a strong enrichment for developmental genes. Moreover, gene set enrichment analysis revealed important cancer-related pathways, including the Wnt and MAPK signaling pathways (Fig. 3e). Then, we examined the relationship between gene expression and combinatorial entropy changes within MM patients, by comparing the expression levels between developmental genes containing hypermethylated eloci in their promoter and bivalent genes without eloci. We found that bivalent genes harboring eloci on their promoter were less expressed but displayed greater interpatient variability (Fig. 3f; only genes with a mean expression level above 1 were taken into account for the coefficient of variation).

Finally, we analyzed the impact of intratumoral methylation variability on developmental genes expression, by separating the genes into two groups according to the PDR level of their promoter (lower or higher than the mean PDR) and calculated, in each group and for each patient, the odds ratio (OR) of the association between gene expression (FPM >1 vs. ≤1) and bivalent promoter methylation (mean methylation < 25% vs. mean methylation > 75%; Fig. 3g; Additional file 7: Table S6). In all MM samples, promoters with a low PDR, i.e., with a greater intra-read homogeneity, tended to maintain the expected opposite relationship between promoter methylation and transcription, whereas in promoters with a high PDR, i.e., with a greater intra-read heterogeneity, for the majority of patients, the link between methylation and expression did not remain significant; for some patients, we even observed a significant link with a relationship opposite to the expected result (OR >1). For example, *LIPG*, which showed comparable methylation levels in two samples (0.61 in M#14 and 0.63 in M#08) coupled with opposite expression levels (FPM of 4.57 in M#14 and 0.29 in M#08), demonstrated the decoupling relationship between promoter methylation and gene expression. M#14 displayed a high promoter PDR (0.80), whereas M#08 displayed a low promoter PDR (0.29; Additional file 3: Figure S20).

Our results demonstrate that disordered DNA methylation gains target the developmental pathway as a whole rather than on specific suppressor genes, and is associated with increased transcriptional variability in MM.

### Combinatorial entropy changes towards hypomethylation preferentially occur in PMDs

Genomic regions that have lost their methylated state, termed partially methylated domains (PMDs), in contrast to “highly methylated domains,” were initially discovered in a fibroblast cell line [62]. Several studies have reported cancer and noncancer human primary cells with PMDs [63–67]. PMDs cover approximately 50 to 75% of the genome of the human primary cell types and tissues investigated, while roughly a quarter are shared, which indicates that PMDs retain strong tissue and cell type specificity characteristics [66]. These domains, which coincide with large-scale regions of repressive chromatin, were recently associated with vast epigenetic changes during carcinogenesis [17]. In this context, we sought to determine whether combinatorial entropy changes, especially hypomethylated eloci found in quiescent states, were related to these genomic regions. For this purpose, we performed a comprehensive analysis of PMDs in MM, using MethylSeekR [23], to analyze WGBS data from our cohort of MM samples and the available WGBS dataset (Additional file 3: Figure S1). We detected PMDs in both NPC samples and in only five MM samples from our cohort (Additional file 3: Figure S21). The PMD structure was highly similar between normal and MM cells despite a very variable level of DNA methylation (Additional file 3: Figure S22a). The base overlap was greater than 80%, the median length distribution was approximately 51 kb, and the mean genome coverage was approximately 65% of the genome (Additional file 3: Figure S22b). Given the very good overlap between NPC and MM PMDs, we subsequently studied PMDs determined from NPCs (referred to as PC-PMDs) in MM patients. As expected, PC-PMD borders were not random and coincided with spatial genome organization, as indicated by the closeness of topological associated domain (TAD) borders to PC-PMD borders, which was larger than expected by chance (p < 0.001). In addition, PC-PMDs shared key features with PMDs of various tumor types and normal tissues [64, 66, 67], including the correlation with lamina-associated domains (LADs) [68], late-replication timing and low gene density (Additional file 3: Figure S23a, b, c). Except for one sample, PC-PMDs that intersected with LADs displayed a late replication time and significantly low methylation levels (Additional file 3: Figure S23d,e). We next examined DNA methylation status across patients. As expected, we found that the DNA methylation level was lower within than outside of PC-PMDs (Additional file 3: Figure S24a) and variable between patients (Additional file 3: Figure S24b). In addition, this variability was associated with the PC-PMD length and replication time (Additional file 3: Figure S25). Notably, PC-PMDs were associated with combinatorial entropy changes as the vast majority of hypomethylated eloci (70.8%) and 26.7% of hypermethylated eloci were located within PC-PMDs. In addition, PC-PMD methylation mean is correlated with an overall epiallele shift increase (Spearman p = 0.46) (Additional file 3: Figure S26). These results support the notion that PC-PMD methylation loss may locally fuel epiallele shifts.

### Severe DNA methylation loss in PC-PMDs is associated with the redistribution of repressive histone marks and perturbations in CGIs/TSSs

**Fig. 4:**
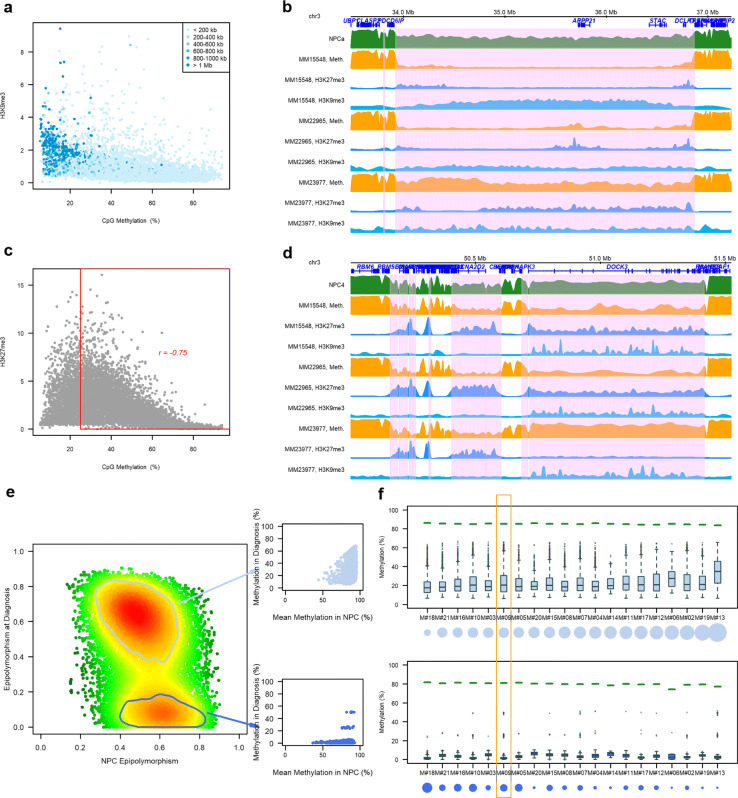
Redistribution of repressive histone marks and combinatorial entropy changes in PC-PMDs (**a**) Average methylation levels inside PC-PMDs according to average H3K9me3 intensity (data from the BLUEPRINT project: ERX1199099 and ERX712768). Each point represents a PC-PMD, and points are colored according to the size of the PC-PMD. (**b**) Example of a long unmethylated PC-PMD (pink area) associated with an increase in H3K9me3 in patient MM15548. (**c**) Average methylation levels in PC-PMDs according to their average H3K27me3 intensity (data from the BLUEPRINT project: ERX1199099 and ERX712769). (**d**) Example of PC-PMDs (pink areas) with methylation loss at diagnosis associated with an increase in H3K27me3 marks. (**e**) Scatterplot of hypomethylated eloci as a function of their epipolymorphism in NPC and diagnosis samples (M#09). The color gradient corresponds to the point density (low is green; high is red). Two eloci populations can be distinguished. The first population (top box) had a moderate decrease in methylation associated with a strong epipolymorphism, and the second population (bottom box) had a low heterogeneity and a drastic methylation decrease at diagnosis. (**f**) DNA methylation levels by patient of eloci with partial demethylation (eloci with an epipolymorphism value between 0.4 and 0.8 in NPCs and remain at the same level in MM) per patient are shown at the top, and DNA methylation levels of eloci with a decreased epipolymorphism value at diagnosis (eloci with an epipolymorphism value between 0.4 and 0.8 in NPCs, and an epipolymorphism value <0.2 at diagnosis) per patient are shown at the bottom. Green segments show the average methylation level of NPCs for these loci. The circles under the patient labels represent the size of the population compared to all hypomethylated eloci. Patient M#19, as an example in Fig. 5e, is outlined in orange

We next sought to study the relationship between DNA methylation and other epigenetic features using available WGBS data together with ChIP-seq data for histone marks (Additional file 3: Figure S1). We found that the DNA methylation losses within PC-PMDs were associated with perturbations in both H3K9me3 and H3K27me3. Notably, the H3K9me3 deposit was associated with severe DNA methylation loss in long PC-PMDs (> 1 Mb) (Fig. 4a), as exemplified for the patient MM15548, with an unmethylated 3 Mb PC-PMD that was highly enriched with H3K9me3 (Fig. 4b). To our knowledge, such widespread H3K9me3 deposits associated with DNA methylation loss have so far been observed only in PMDs of cancer cell lines [35, 66, 69]. H3K27me3 was also perturbed inside PMDs. Notably, H3K27me3 enrichment was correlated with DNA methylation erosion (Fig. 4c). One notable example is the *DOCK3*-containing PC-PMD, which is enriched in H3K27me3 and showed H3K27me3 deposits in patients MM15548 and MM22965, who also exhibited DNA methylation erosion in this PMD; however, in another patient (MM23977) with a methylation level in this PMD comparable to that in NPCs, the H3K27me3 mark was absent (Fig. 4d). Taken together, these results show that perturbations in repressive histone marks are variable and depend on genomic regions, patients and DNA methylation levels.

**Fig. 5:**
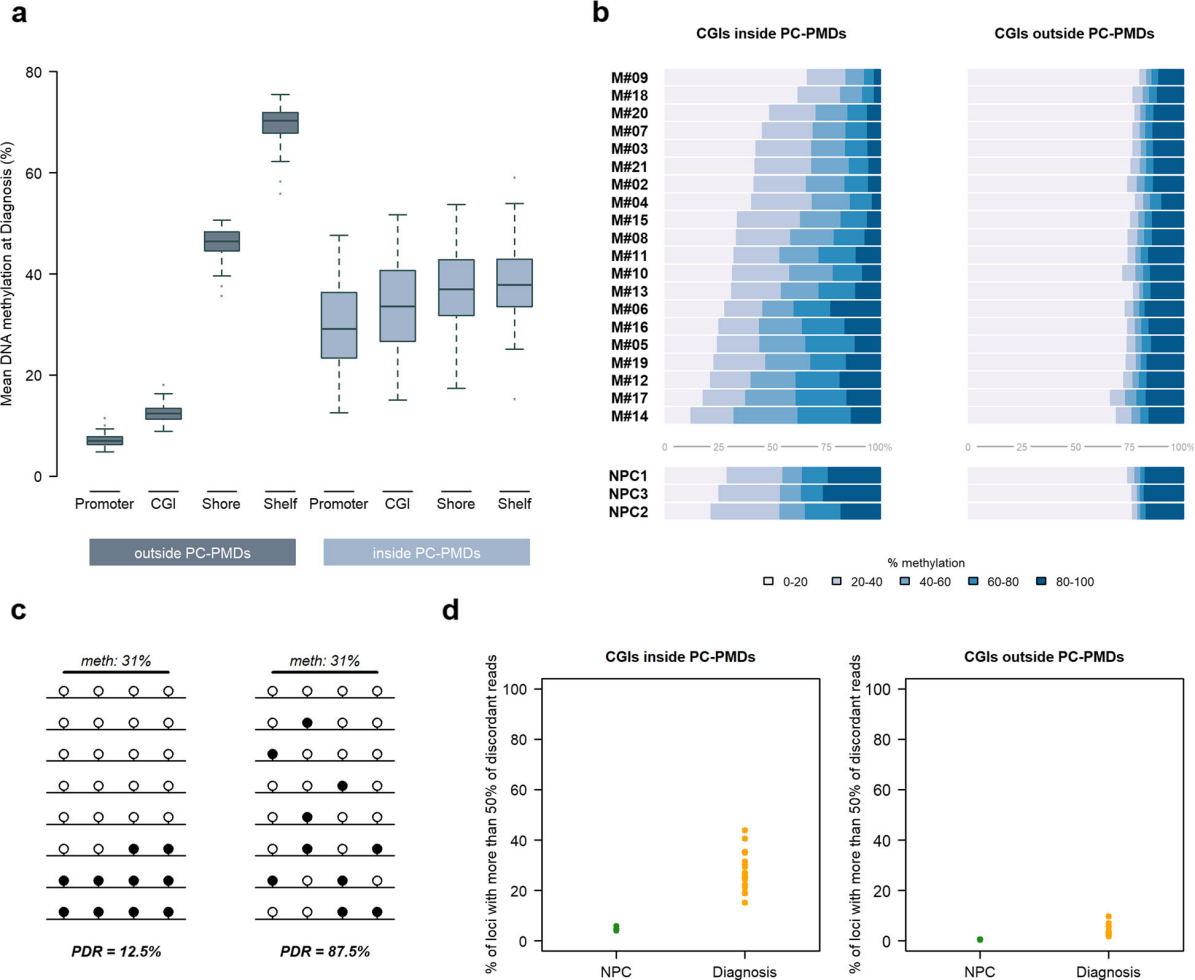
Intermediate methylation level of CGIs in PC-PMDs. **a** Mean DNA methylation level at diagnosis in promoter, CGIs, shores, and shelves regions according to the presence or absence of PC-PMDs. **b** Proportion of CGIs according to their methylation levels inside and outside PC-PMDs: horizontal bars represent individual samples (MM at the top and NPC at the bottom). **c** Example of a locus with the same average level of DNA methylation and radically different PDR values. **d** Proportion of loci with more than 50% discordant reads inside and outside PC-PMDs

Given that some regions are hypomethylated in MM compared to NPCs while others are completely demethylated, we next studied combinatorial entropy changes that occurred in these domains using the hypomethylated eloci obtained with eRRBS. We identified in most of the MM samples two subsets of hypomethylated eloci compared to normal samples (Fig. 4e,f; Additional file 3: Figure S27). The first type included extensively demethylated loci (Fig. 4f, bottom) and the second type contained epipolymorphic loci emerging due to partial methylation loss (Fig. 4f, top). These eloci were variable depending on the patient. Collectively, this suggests that in some genomic regions, combinatorial entropy changes are related to severe DNA methylation loss, which could be replaced by H3K9me3, whereas some domains are characterized by predominant stochastic DNA methylation loss, reflected by high epipolymorphism associated to intratumor heterogeneity. These domains would be more associated with a redistribution of the H3K27me3 mark.

We next investigated the impact of PC-PMD methylation loss on regulatory regions. We found that the normal near-bimodal methylation state, observed outside of PC-PMDs, was completely abolished inside PC-PMDs (Fig. 5a). As a result, promoter CGIs lost their hypomethylated state and gained intermediate methylation levels, while adjacent regions became less methylated to reach an intermediate degree of methylation. Notably, the acquisition of DNA methylation inside PC-PMDs resulted in a significant increase in the intermediate methylation state of CGIs at the expense of strictly methylated or unmethylated states in all MM patients examined (Fig. 5b), in agreement with results obtained in breast cancer [64]. Interestingly, this phenomenon was also observed, albeit to a lesser extent, in NPCs (Fig. 5b). As identical average methylation may correspond to uniform or disordered states (Fig. 5c), we sought to discriminate between both states by calculating the PDR. We showed that the percentage of loci with a high level of discordant reads (> 50%) was higher in all MM compared to control patients, indicating that although PC-PMD methylation is disturbed in NPCs, CGI methylation patterns are more homogeneous (Fig. 5d). Interestingly, a large proportion of bivalent promoter CGIs with disrupted DNA methylation was embedded within PC-PMDs (39% totally included and 54% with at least 30% of shared bases, as exemplified in Additional file 3: Figure S28).

**Fig. 6:**
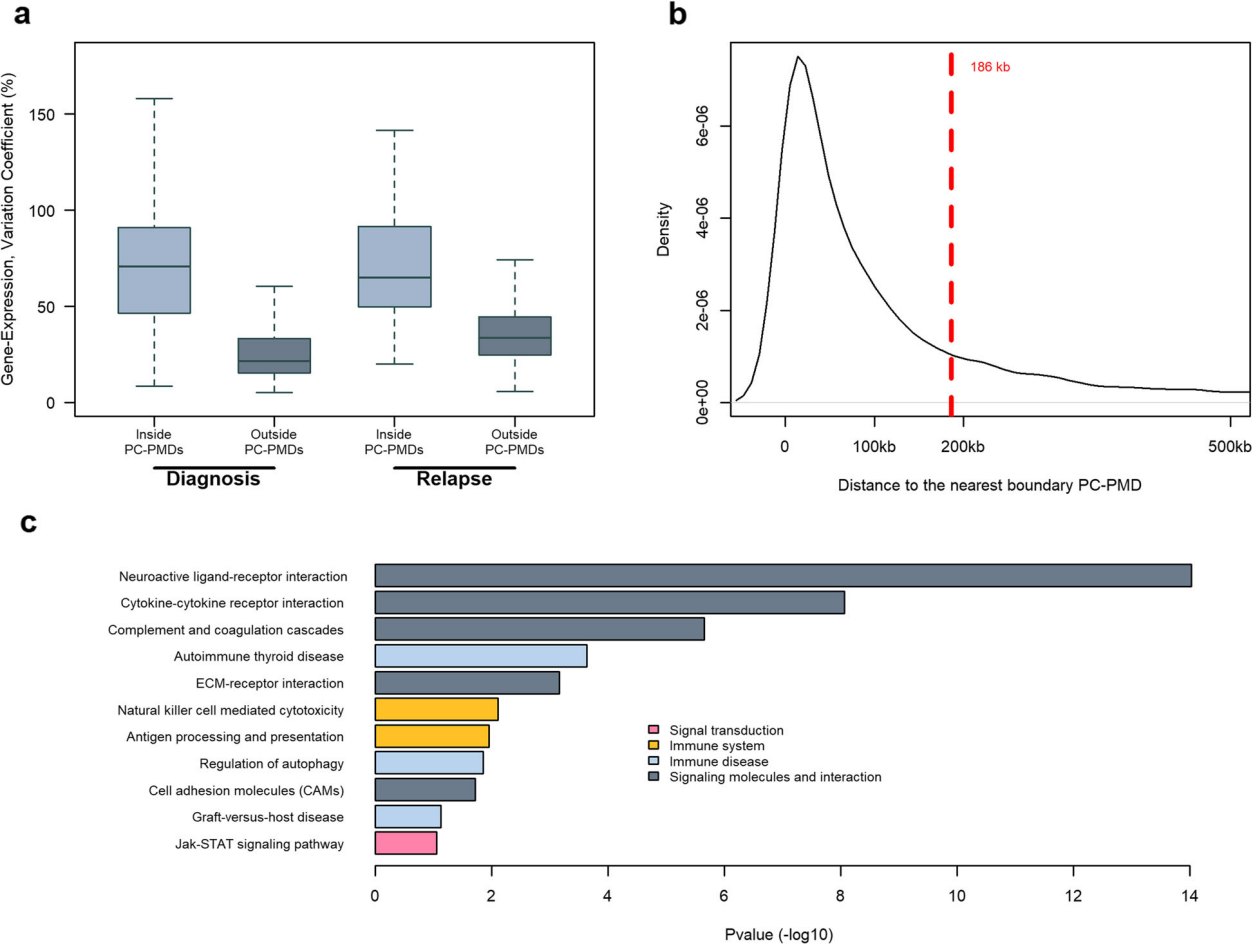
Gene expression variability in hypomethylated PC-PMDs. **a** Variation coefficient of gene expression in PC-PMD and non-PMD regions at diagnosis (left) and relapse (right). **b** Density of the TSS distance to the nearest PC-PMD boundary. The red dotted line corresponds to the 3rd quartile of the TSS distance to the nearest PC-PMD boundary. **c** Ontological analysis of genes in PC-PMDs located less than 186 kb from a PC-PMD boundary

Given the interpatient heterogeneity of PMD methylation, we also investigated variations in gene expression. We found more gene expression variability inside PC-PMDs than outside of PC-PMDs; this difference was also maintained at relapse (Fig. 6a). Interestingly, we found that genes inside PC-PMDs were abundant within 186 kb of PC-PMD boundaries (Fig. 6b). We therefore focused our analysis on genes located near boundaries: Kyoto Encyclopedia of Genes and Genomes (KEGG) pathway analysis revealed specific enrichment in immune-related pathways including cytokine-cytokine receptor interaction, complement and coagulation cascades, autoimmune thyroid disease, natural killer cell-mediated cytotoxicity, antigen processing and presentation, regulation of autophagy, cell adhesion molecule, and graft-versus-host disease (Fig. 6c). This enrichment pattern in PC-PMDs could provide an explanation for immune evasion mechanisms that occur during tumor progression [70, 71].

Taken together, these results show that MM displays a high epigenomic instability and great transcriptomic variability in PC-PMDs associated to high combinatorial entropy changes in some regions, which might be beneficial for tumor cells.

### 3D genome architecture reorganization is favored in hypomethylated PMDs

**Fig. 7:**
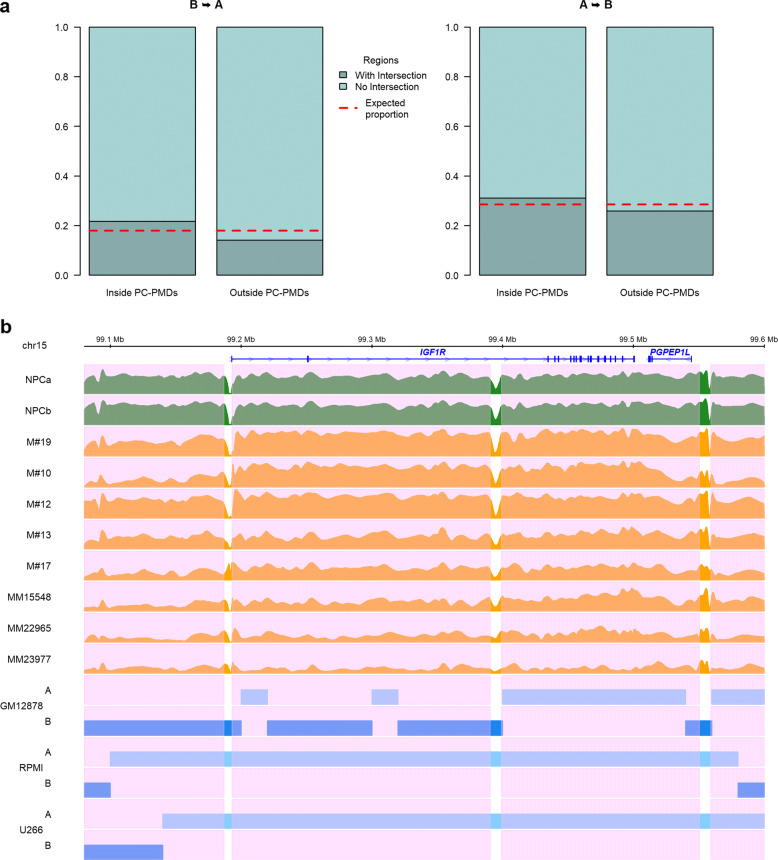
Hypomethylated PC-PMDs and the reorganization of compartment A/B. **a** Barplots illustrating the proportion of regions switching from compartment B to A (left) and from A to B (right) intersecting with or without a PMD region. The red dotted lines correspond to the expected proportions. **b** Example of *IGF1R* gene locus switching from compartment B to A (GM12878 cell line versus MM cell lines). Pink areas indicate PC-PMD regions

Global DNA methylation loss impacts spatial genome organization in the nucleus [72]. We wondered whether severe DNA hypomethylation that occurs during MM development could reshape 3D chromatin architecture in MM cells. The chromatin fiber of eukaryotic genomes is folded at multiple levels, including large-scale genomic structures to form distinct chromatin compartments A and B, characterized by gene-dense transcriptionally active open chromatin and gene-sparse transcriptionally closed chromatin, respectively [73]. During cancer development the genome is reorganized, and as a consequence, genomic regions of compartment A switch to those of compartment B and vice versa. The proportion of compartment A/B switching varies according to tumor type [74, 75]. We investigated the relationships between PC-PMDs and compartment A/B switching. We determined compartment A/B boundaries at 20-kb resolution from Hi-C data in a lymphoblastoid B cell line (GM12878), with a DNA methylome similar to that of PCs [76], and in the myeloma cell lines U266 and RPMI8226. We found that PC-PMDs were more prone to switch than other genomic regions (Fig. 7a). As a representative example, *IGF1R*, which encodes a major mediator of growth and survival in MM, and is located astride compartments A and B in NPCs, switched entirely to compartment A in both myeloma cell lines (Fig. 7b).

Altogether, these results show that compartment switching might be favored by PMD instability.

## Discussion

MM is a heterogeneous disease. However, until now, only studies on genetic abnormalities have demonstrated the presence of subclonal populations that encountered complex evolution during the course of a disease. A recent study highlighted the contribution of intratumor epigenetic heterogeneity to shape chronic lymphocytic leukemia (CLL) evolution after therapy [77]. To assess intrapatient DNA methylation heterogeneity in MM, we used the eRRBS technique, which allows us to capture the methylation status of individual CpGs in a single cell (more precisely in one single allele), with a large sequencing depth. Combining various DNA methylation heterogeneity metrics, we showed that the MM epigenome is characterized by high intratumor heterogeneity with predominantly stochastic methylation patterns.

The fact that the acquisition at the time of diagnosis of a higher EPM confers a poor survival outcome independently of high-risk genetic lesions in our modest cohort provides strong arguments to validate the prognostic impact of epiallele shifting in newly diagnosed MM patients enrolled in phase 3 trials.

We found that loci that undergo epiallele shifting associated with a gain of DNA methylation are enriched within bivalent promoter-associated chromatin states and target developmental genes. De novo DNA methylation of bivalent promoter has already been reported in many other cancer cell lines and primary tumors and more recently during resetting of hESCs to the naïve state [60, 61, 78]. We demonstrated that in MM these DNA methylation gains are associated with high heterogeneity, in agreement with results obtained in acute myeloid leukaemia [18]. The purpose of hypermethylation of a large number of loci targeting developmental genes remains poorly understood and a topic of debate. To this end, a study has recently established a functional link between the hypermethylation of the CGI promoter of these genes and oncogenic transformation, demonstrating a causal relationship between the hypermethylation and the acceleration of the transformation [79]. Interestingly, in a model wherein hematopoietic progenitors are proposed to be the cells of origin in MM, an aberrant epigenetic program persisting through normal cell differentiation is implicated in tumor initiation [80]. Further analysis of this aberrant DNA methylation program revealed strong enrichment of biological functions associated with developmental regulation (Additional file 3: Figure S29), suggesting that a disruption in developmental pathways does not prevent differentiation into plasma cells but could play a key role in the initiation of MM and increase susceptibility to oncogene transformation in response to environmental changes [81]. Moreover, considering the link between age and cancer predisposition demonstrated by Tao et al. [79], we can assume that disturbance of developmental genes in MM is partially age-related. This warrants further investigation particularly in pre-malignant condition.

During normal development and differentiation, these genes are not regulated by the presence of promoter DNA methylation but by repressive histone modifications [82], which allows low basal transcription states that are dynamically inducible to ensure balance between stem cell self-renewal and lineage/differentiation. We demonstrate that gains of DNA methylation that we observed in promoter of developmental genes in MM are associated with an elevated PDR, which leads to a decoupling relationship between promoter methylation and transcription. These results are in line with those obtained in CLL [19]. Single cell RNA-seq analysis showed that a high PDR is correlated with a “noisy” transcriptional landscape and an intermediate transcriptional state that interferes with complete silencing or high-level expression [19]. Several studies have also shown the role of cellular heterogeneity and gene expression noise in overcoming drug resistance or metastatic barriers [83–85]. Together, these data suggest that increased stochastic methylation variation allows tumor cells to better adapt and find new trajectories in response to environmental changes or under treatment pressure.

A large majority of combinatorial entropy changes between normal and malignant plasma cells associated with DNA methylation loss are found in regions that are already partially methylated in normal cells, indicating that PC-PMDs delimit genomic regions in which methylation information is not accurately maintained due to reduced energy consumption and channel capacity, as recently demonstrated for compartment B [17]. These large regions that cover approximately 70% of the genome are the major source of variability within MM patients.

In myeloma cells, PC-PMD hypomethylation is associated with other key epigenetic aberrations, such as promoter CGI hypermethylation. Indeed, we observed a loss of the hypomethylated state and gained intermediate methylation levels in regulatory regions within PC-PMDs. This disturbance has already been observed in NPCs; however, disordered methylation at promoters is lower in NPCs than in malignant cells, indicating a homogeneous methylation pattern at the promoter being either fully methylated or unmethylated.

Approximately half of bivalent promoters with a high PDR in MM are associated with PC-PMDs. Therefore, the DNA methylation landscape of MM resembles that of the placenta, with stochastic methylated gain in CGIs embedded in large hypomethylated regions, suggesting that, as in other cancers, myeloma cells coopt placental nuclear programming [86, 87]; this finding is of particular interest since placental and cancerous tissues share relevant features such as immune modulation, angiogenesis, and tissue invasion [88]. Interestingly, the specific enrichment of genes in immune-related pathways was revealed when we focused our analysis on genes located near PC-PMD boundaries.

PC-PMDs are also linked to the redistribution of repressive marks. We showed that PC-PMD hypomethylation is associated with a disruption in the H3K9me3 and H3K27me3 marks and variable depending on genomic regions, patients, and DNA methylation levels. According to the model of Reddington et al. [89], we can hypothesize that DNA methylation prevents *PRC2* from binding to inappropriate targets, and that global hypomethylation due to tumorigenesis drives H3K27me3 redistribution responsible for the derepression of target genes and the repression of new genes. This redistribution may partially explain the derepression of *HOXA9* [90] and the de novo bivalent promoters [91] observed in MM. H3K27me3 redistribution could also play an important role in chromatin decompaction, as has been shown in ESCs [92], and could explain the increase in open chromatin regions within the heterochromatin state of MM samples [93]. These data highlight that PMDs must be considered as a separate entity in genomic analyses.

Finally, we showed that DNA hypomethylation is variable within the myeloma cells of one individual and that methylation loss modifies H3K27me3 distribution across patients; we can hypothesize that H3K27me3 redistribution is heterogeneous among myeloma cells, leading to cell-to-cell epigenetic variability. Consequently, the tumoral mass in a MM patient would be composed of an admixture of myeloma cells with divergent epigenetic identities, in accordance with what has been demonstrated recently in CLL [94].

We cannot rule out the possibility that other genomic regions, particularly active enhancers, are also associated with intratumor heterogeneity in MM as it was previously shown in two other hematological malignancies [18, 94]. A comprehensive analysis using WGBS combined with analysis methods that encapsulate underlying epigenetic variability and patient-specific histone marks could address this question.

## Conclusions

Altogether, our results show the importance of genome-wide epigenetic analysis and reveal marked PMD instability in MM patients at presentation. The perturbation in PMDs occurs at the epigenetic, transcriptomic, and 3D organization levels and is responsible for interpatient variability. As numerous studies showed that hypomethylation is associated to genomic instability [14, 95] and given that genetic stability was altered within breast PMDs [64], we can assume that PC-PMDs are domains in which instability at the genetic level is also tolerated. This disturbance in PMDs could explain, in part, some aberrant and heterogeneous phenotypes across MM samples [96, 97]. In addition, the lack of accurate global DNA methylation maintenance also drives intrapatient DNA methylation heterogeneity, which can contribute to intrapatient variability, allowing cell-to-cell diversity in transcriptional programs and opening multiple trajectories in response to therapy (Additional file 3: Figure S30).

Finally, our results confirm that DNA methylation is already disturbed in NPCs but yet remains greatly homogeneous with mostly uniform reads. We can assume that accumulation of aberrant DNA methylation coupled with genetic alterations at the premalignant stages could reach a “point-of-no-return” which would lead to a hypomethylated malignant stage associated with intratumor epigenetic heterogeneity and a mixture of genetic subclones.

## Supplementary Information

**Additional file 1** Table S1. Data resource.

**Additional file 2** Table S2. Description of sequencing metrics for eRRBS data.

**Additional file 3** Figures S1–S30. Supplementary figures.

**Additional file 4** Table S3. Distribution of CpG sites within 100 bp genomic units in the human genome.

**Additional file 5** Table S4. Average number of CpGs covered by eRRBS with read depths greater than 10X and 60X, given by genomic features.

**Additional file 6** Table S5. Multivariable Cox regression models for RFS including EPM along with the 5 high-risk chromosomal changes (a) all variables in a single model, (b) stepwise method.

**Additional file 7** Table S6. Odds ratios and 95% confidence intervals for association between gene expression and promoter methylation according to low or high PDR levels in the promoter.

## Data Availability

SNParray, WGBS, eRRBS, and RNA-seq data have been deposited at the European Genome-phenome Archive (EGA, https://www.ebi.ac.uk/ega), which is hosted by the EBI and the CRG, under dataset accession EGAD00010002102 (https://ega-archive.org/datasets/EGAD00010002102), EGAD00001007821 (https://ega-archive.org/datasets/EGAD00001007821), EGAD00001007822 (https://ega-archive.org/datasets/EGAD00001007822), and EGAD00001007813 (https://ega-archive.org/datasets/EGAD00001007813), respectively. WGBS Blueprint data of normal plasma cells and multiple myeloma patient are available from the EGA under the accessions EGAD00001002322 (https://ega-archive.org/datasets/EGAD00001002322) and EGAD00001002521 (https://ega-archive.org/datasets/EGAD00001002521) respectively [22, 53, 76]. ChIP-Seq Blueprint data of multiple myeloma patient are available from the EGA under the accessions EGAD00001002379 (https://ega-archive.org/datasets/EGAD00001002379) [22].

## Abbreviations

3D: Three-dimensional
bmPC: Plasma cell from bone marrow
CGI: CpG island
CLL: Chronic lymphocytic leukemia
DAVID: Database for annotation, visualization and integrated discovery
eloci: Epigenetic loci
EPM: Eloci per million loci covered
eRRBS: Enhanced reduced representation bisulfite sequencing
ESCs: embryonic stem cells
FDR: False discovery rate
FPM: Mapped read counts per million of mapped fragments
GC: Germinal center B cell
HPC: Hematopoietic progenitor cell
ICED: Iterative correction and eigenvector decomposition
IFM: Intergroupe francophone du myélome
KEGG: Kyoto Encyclopedia of Genes and Genomes
LAD: Lamina-associated domain
memBC: Memory B cell
MGUS: Gammopathy of undetermined significance
MM: Multiple myeloma
NBC: naïve B cell
NPCs: Normal plasma cells
OR: Odd ratio
PCs: Plasma cells
PDR: Proportion of discordant reads
PMDs: Partially methylated domains
preBC: Precursor B cell
RFS: Relapse-free survival
RNA-seq: RNA sequencing
RRBS: Reduced representation bisulfite sequencing
SMM: Smoldering multiple myeloma
SNPs: Single-nucleotide polymorphism
TAD: Topological associated domain
tPC: Plasma cell from tonsil
TSS: Transcription start site
TTS: Transcription termination site
WGBS: Whole genome bisulfite sequencing
