# Supplementary material for: The DNA methylation landscape of multiple myeloma shows extensive inter- and intrapatient heterogeneity that fuels transcriptomic variability
Source: Genome Med. 2021 Aug 9;13:127. doi: 10.1186/s13073-021-00938-3 (PMC8351364; doi:10.1186/s13073-021-00938-3)
Supplement: Supplementary file 3 — Additional file 3 Figures S1–S30. Supplementary figures. [file 13073_2021_938_MOESM3_ESM.docx]

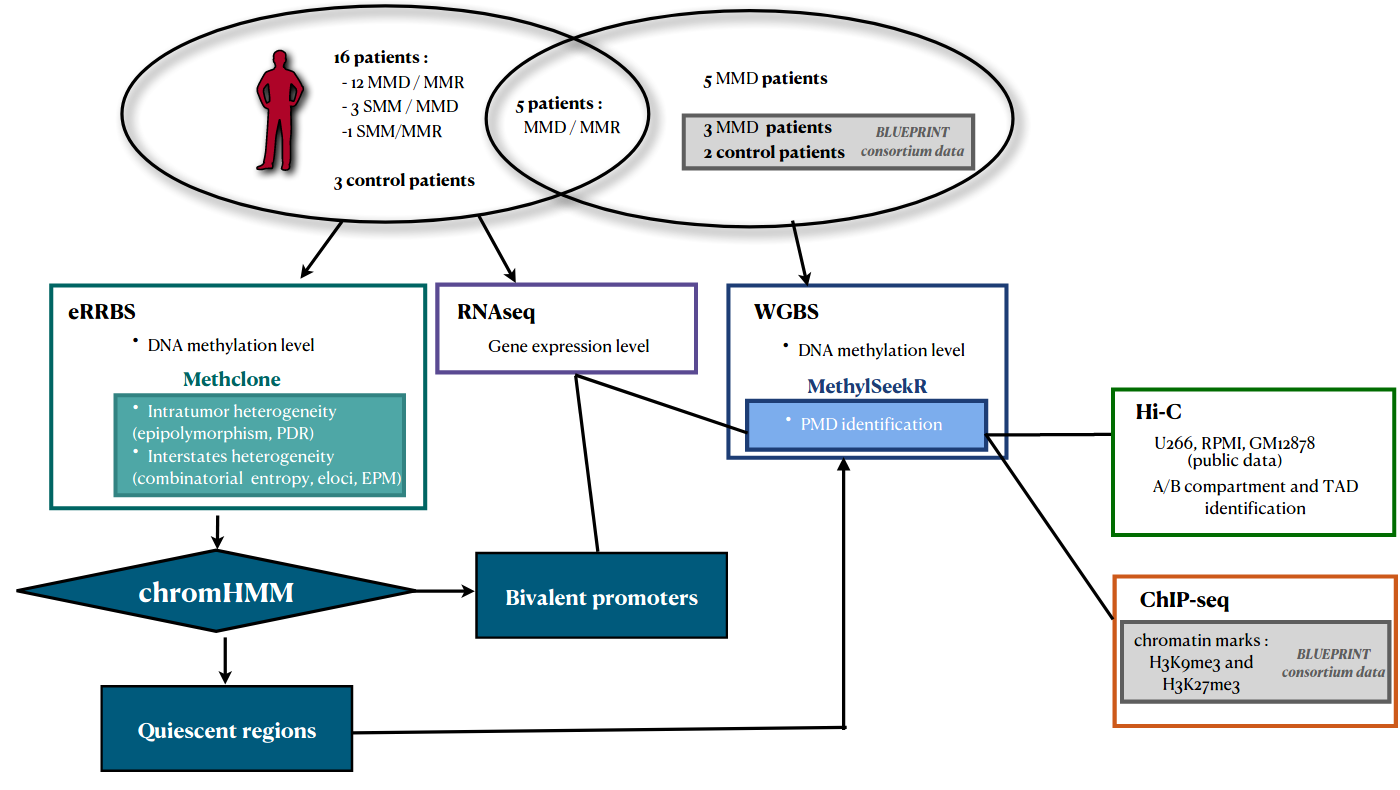


**Figure S1 |** Diagram of the analytical process


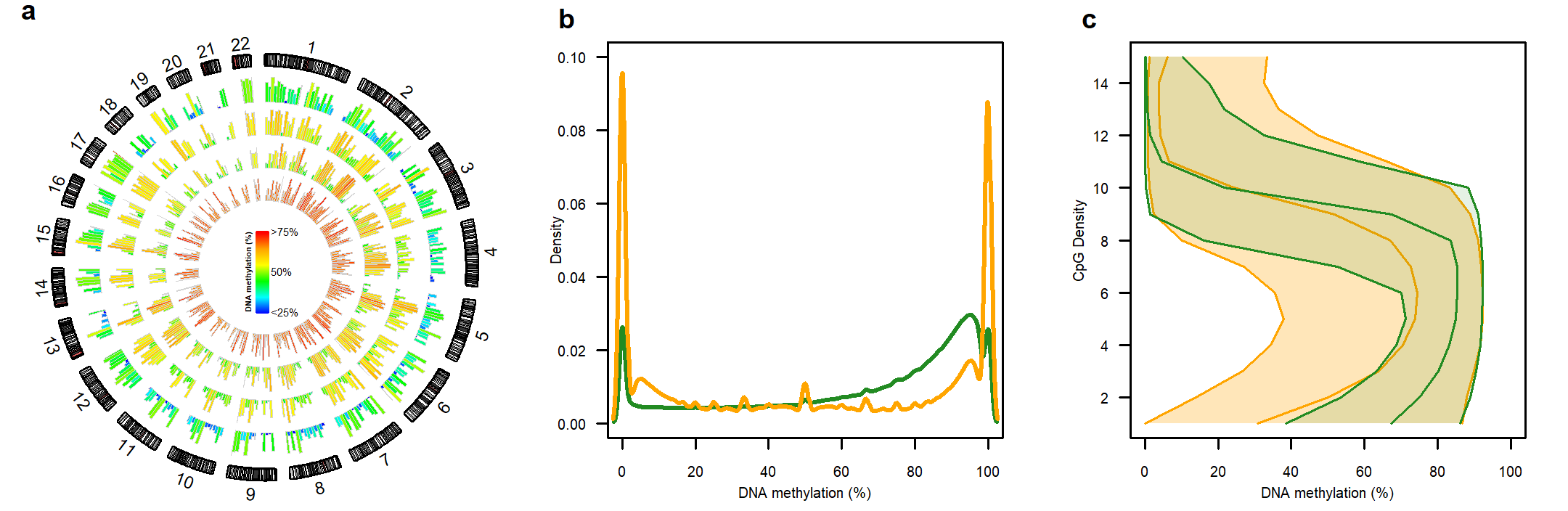


**Figure S2 |** Global analysis of the DNA methylome of myeloma samples at diagnosis and NPC samples. (a) Circos plot of DNA methylation levels measured in WGBS data. From the center to the outside: NPCb (data from the BLUEPRINT project, ERX301127), M#17, M#19, and M#10. Histograms represent CpG methylation levels averaged in 10-Mbp genomic windows. (b) Density distribution of DNA methylation levels measured in WGBS data for NPC (green, data from the BLUEPRINT project, ERX301127, ERX715130) and MM diagnosis (orange) samples. This color code was used in all figures. (c) CpG methylation within 100 bp genomic units as a function of local CpG density.

**
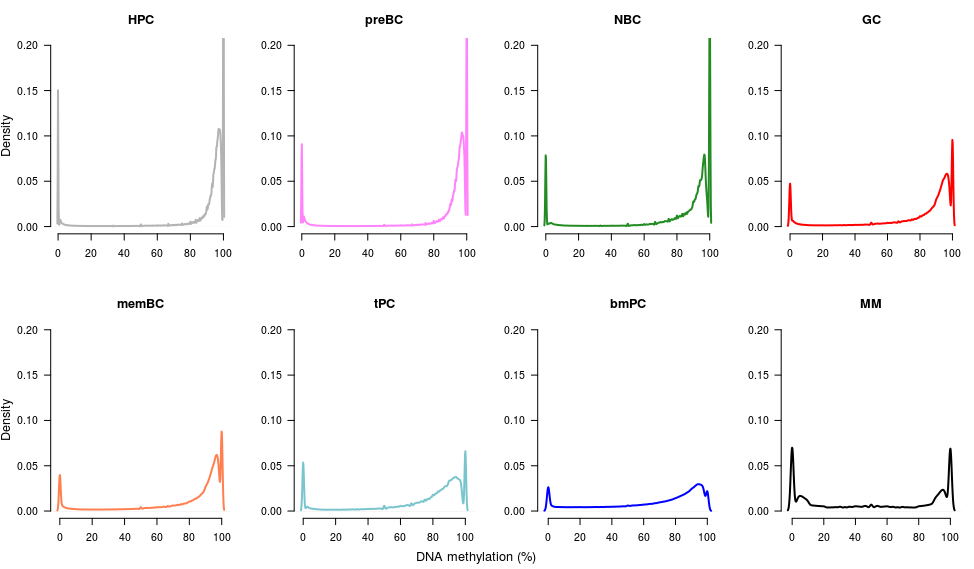
**

**Figure S3 |** Density distribution of DNA methylation levels for hematopoietic progenitor cell (HPC), precursor B cell (preBC), naïve B cell (NBC), germinal center B cell (GC), memory B cell (memBC), plasma cell from tonsil (tPC), plasma cell from bone marrow (bmPC) and MM samples measured by WGBS.


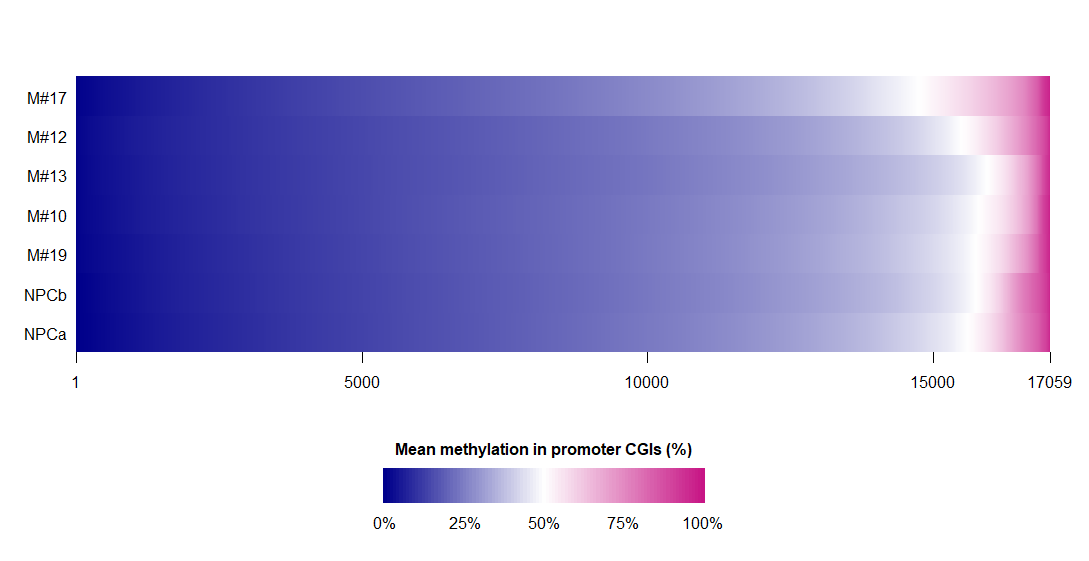


**Figure S4 |** Mean methylation levels in promoter-CGIs across MM patients and NPCs.


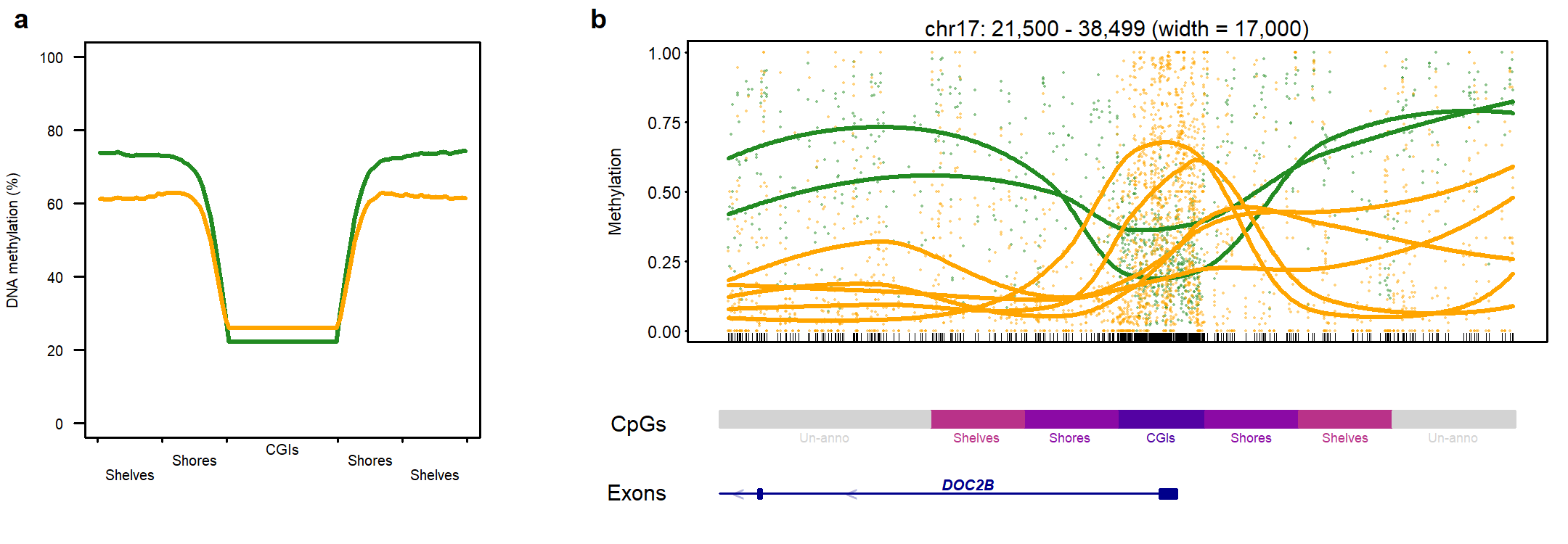
**Figure S5 |** Disrupted methylation in CpG-islands and flanking regions (a) Mean methylation of CGIs and flanking regions in MM patients compared to NPCs. (b) Dispersive DNA methylation surrounding the *DOC2B* promoter in MM samples and in NPCs.


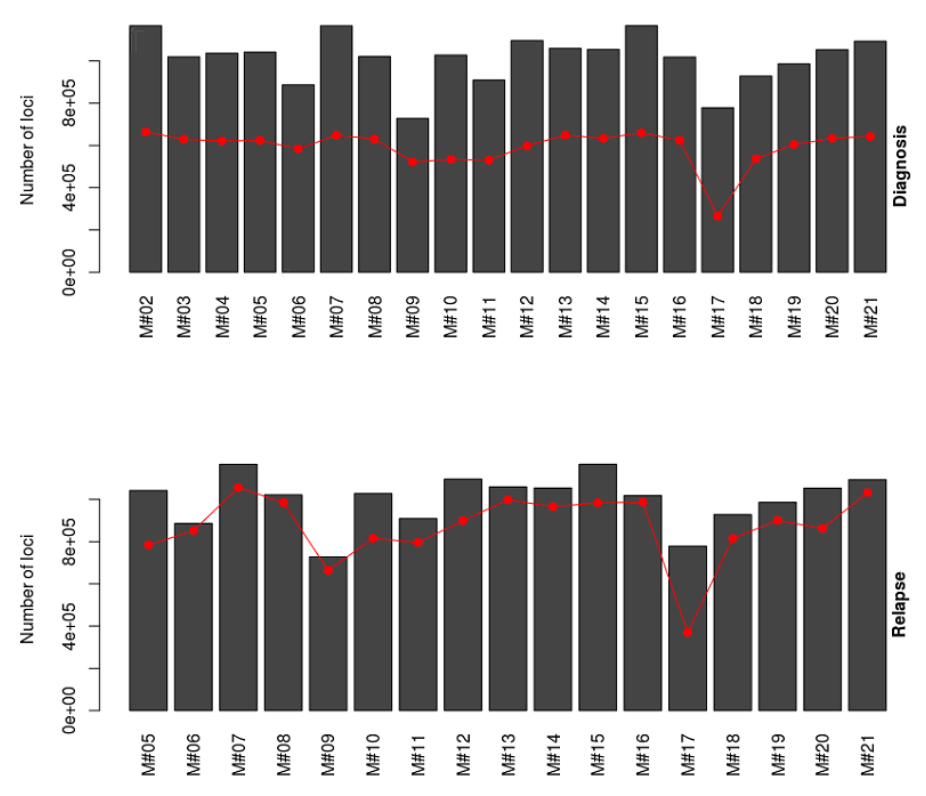
**Figure S6 |** Number of loci sequenced per diagnosis (top) and relapse (bottom) samples. The red dots

correspond to the number of comparable loci between NPCs and MM patient samples at the top and

between samples at diagnosis and relapse at the bottom.


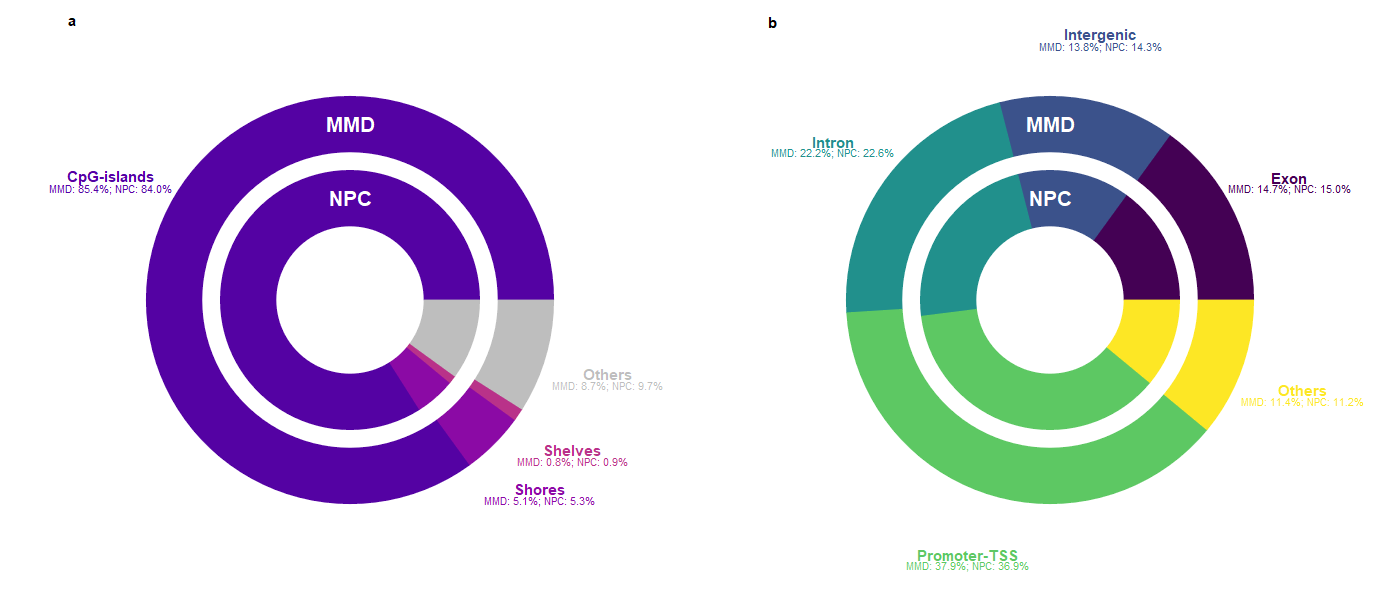


**Figure S7 |** Genome distribution of background loci across MM patients and NPCs in CpG islands, shores and shelves (a), and in promoter TSS, intron, exon and intergenic regions (b).


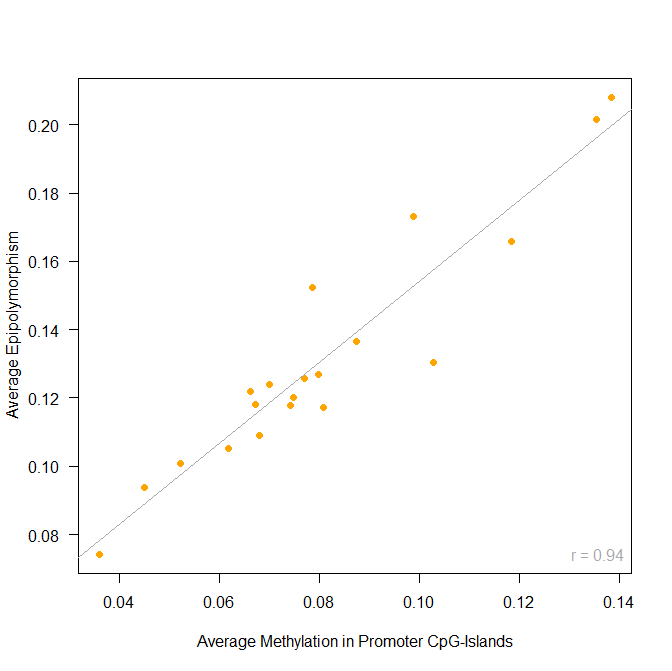


**Figure S8 |** Correlation between average promoter CGI methylation and epipolymorphism at diagnosis per sample.


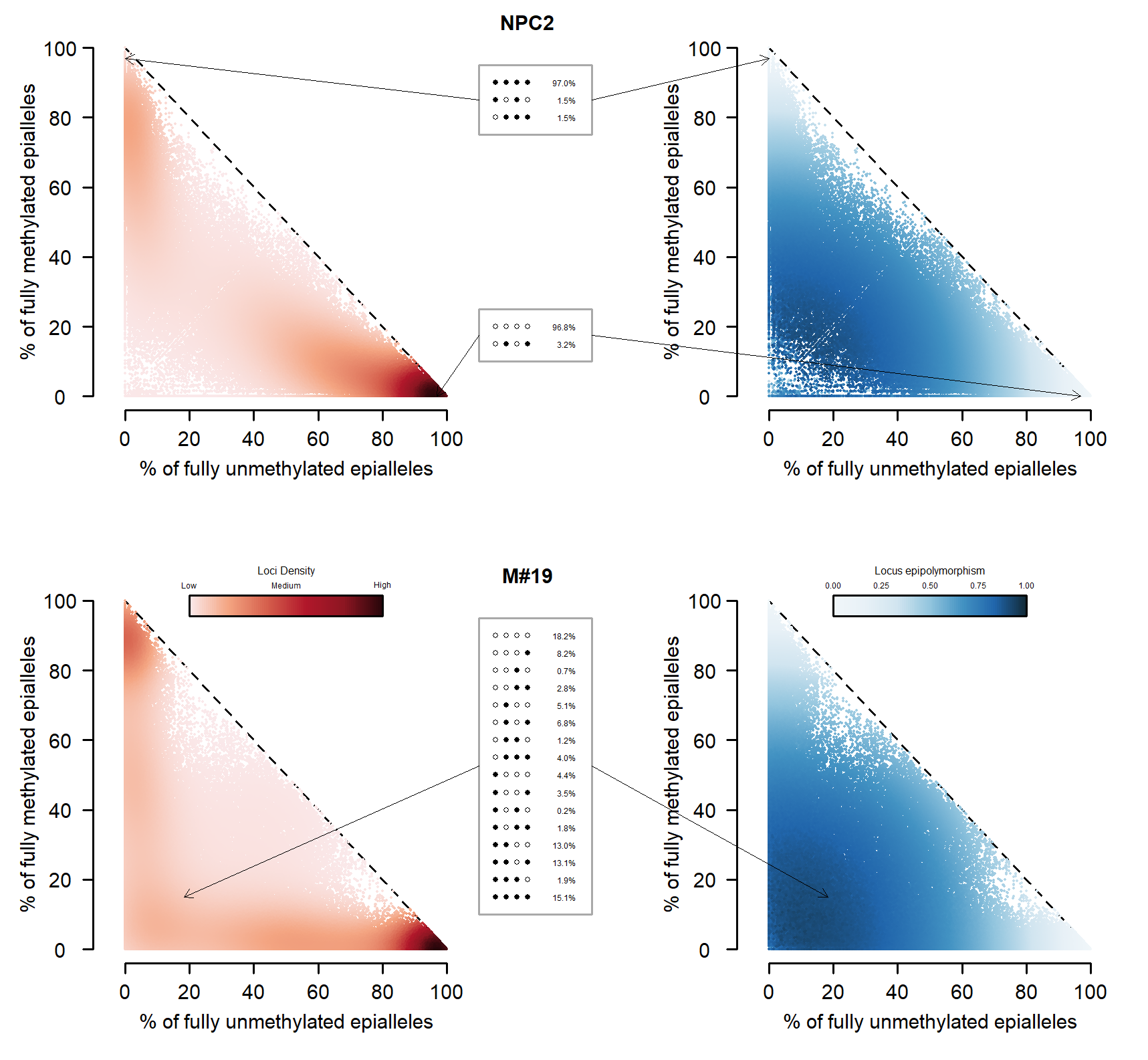


**Figure S9 |** Scatterplots showing loci organization for NPC2 and M#19 diagnosis samples. Each point corresponds to a locus of 4 adjacent CpGs. On the left, each point is color coded according to the density of the surrounding points; on the right, each point is color coded according to its epipolymorphism level.


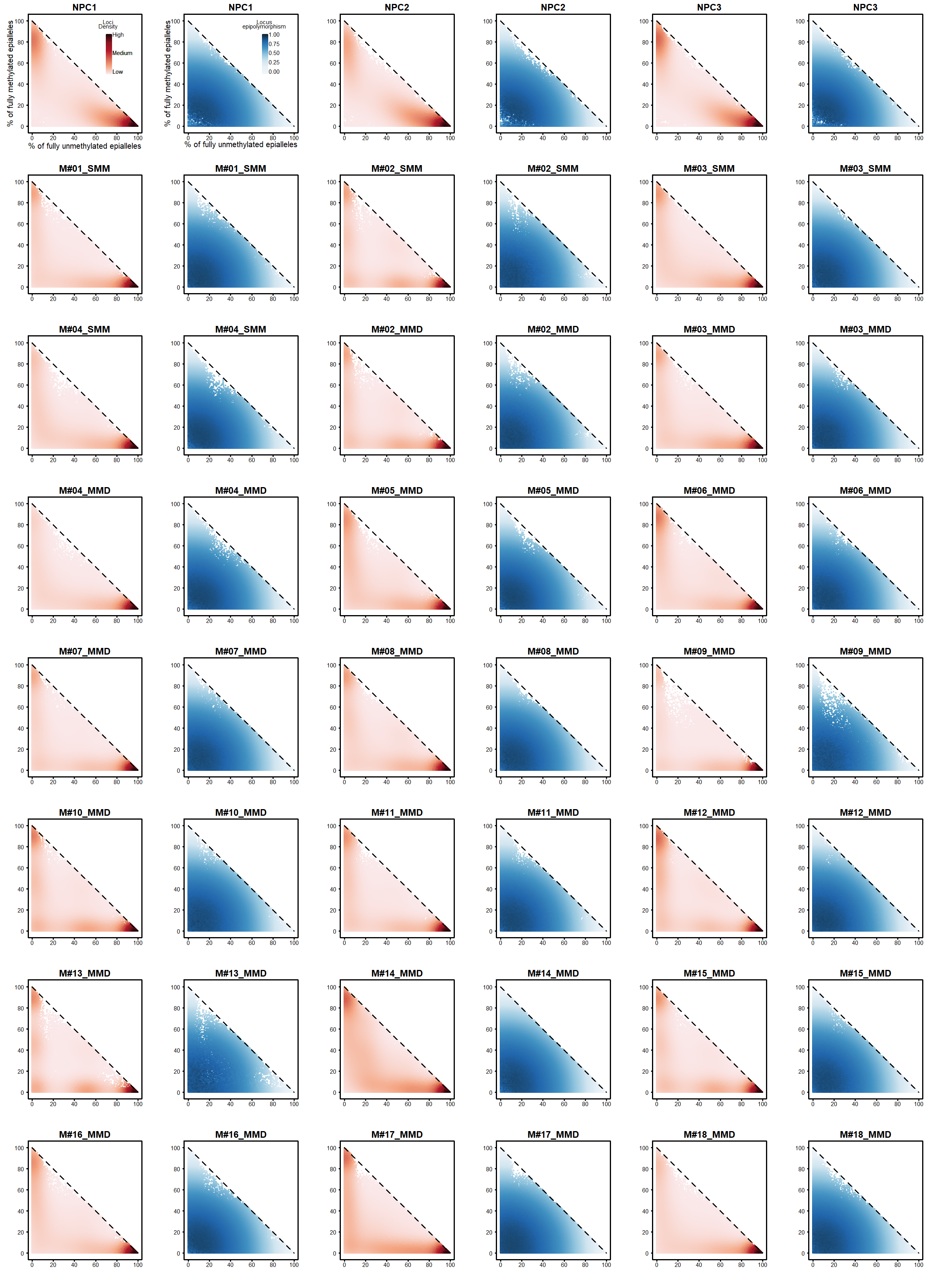


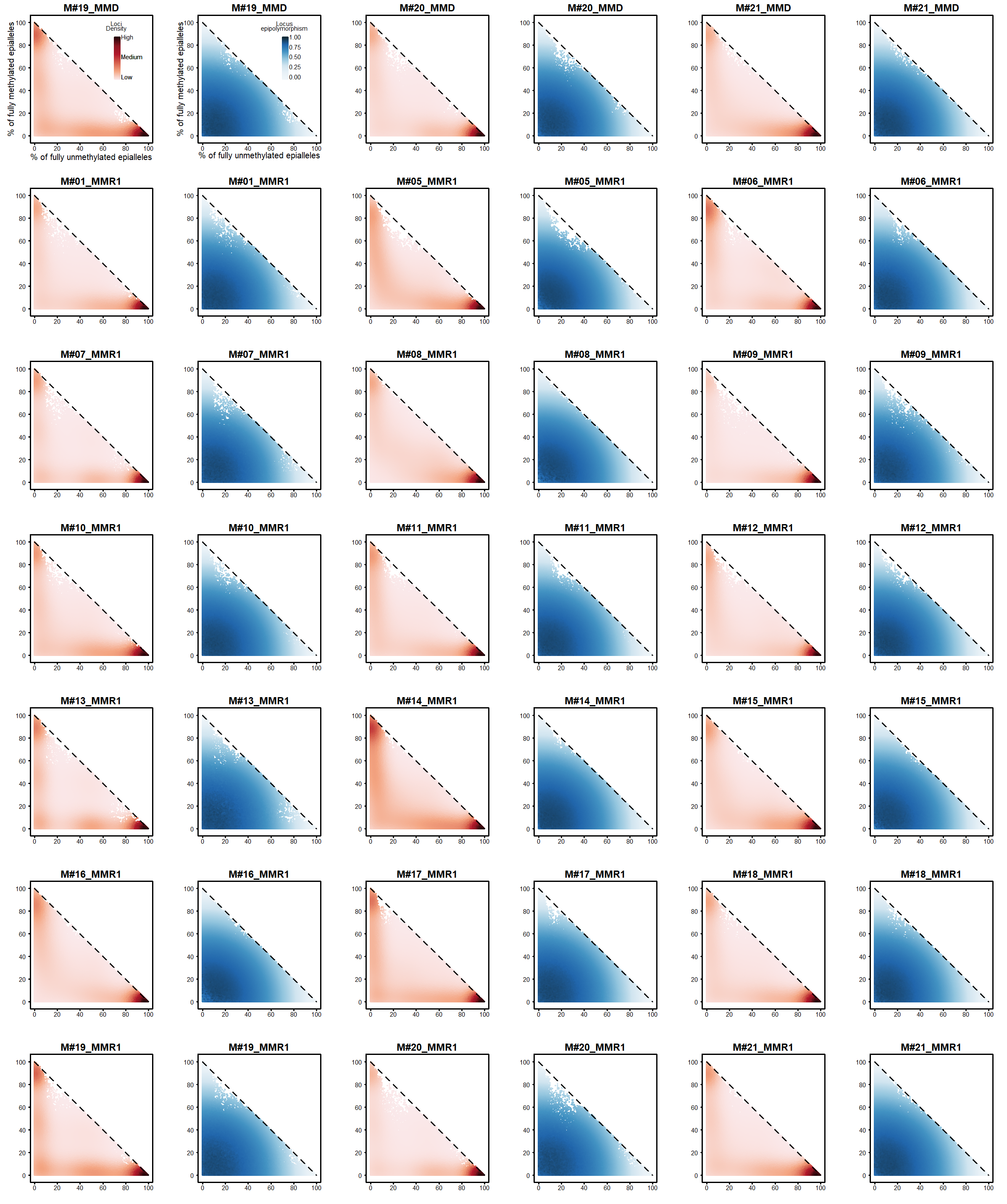


**Figure S10 |** Scatterplots showing loci organization as in Figure 2b for NPCs and all paired samples (SMM/Diagnosis/Relapse). Each point corresponds to a locus of 4 adjacent CpGs. Shown on the left is a representation where each point is color coded according to the density of the surrounding points; on the right each point is color coded according to its level of epipolymorphism.


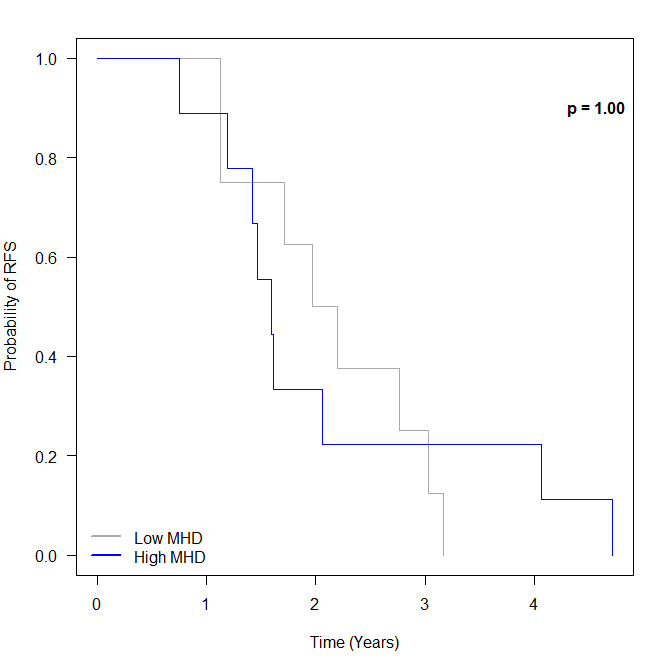


**Figure S11 |** Time to relapse analysis for patients according to intratumor methylation heterogeneity (MH) values: low (below median value; grey) *vs*. high (above median value; blue).


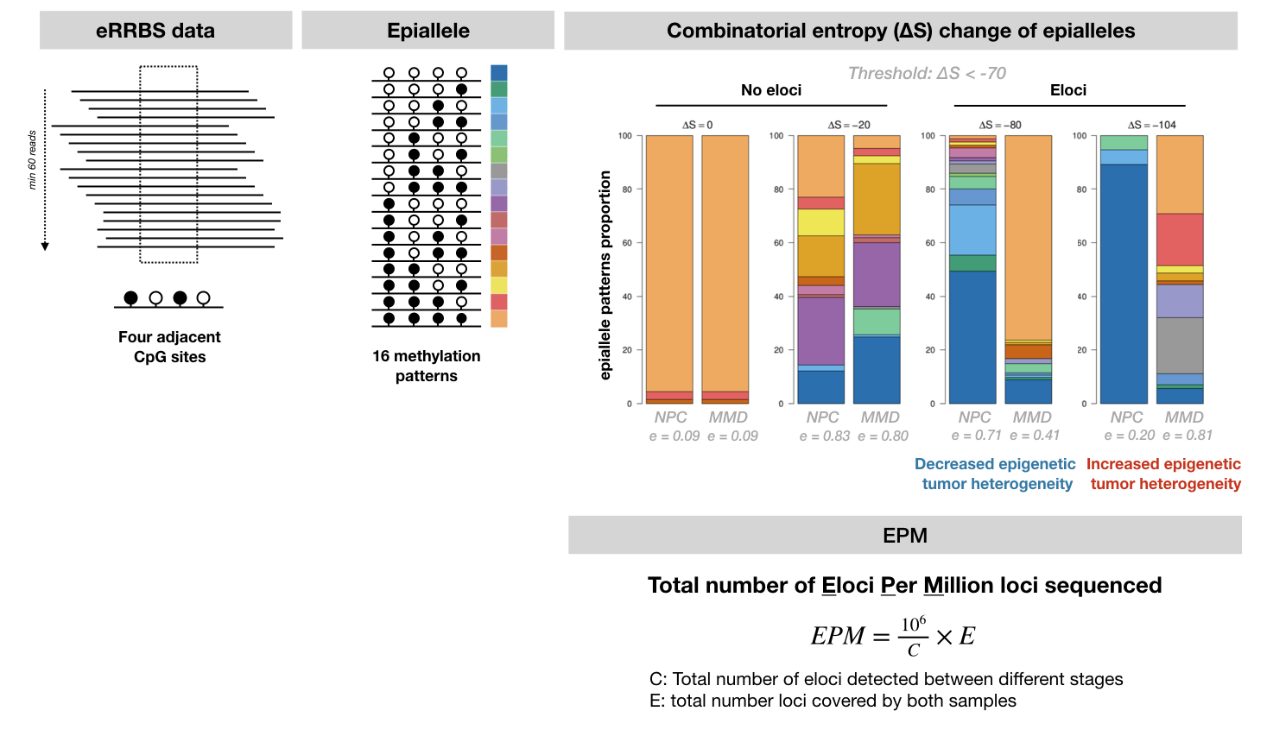


**Figure S12 |** Detection of epiallelic changes. From a combinatorial entropy value (threshold: ∆S <-70), Methclone determines loci with a significant change in epiallelic composition (= eloci). Filled black circle: methylated CpG; empty circle: unmethylated CpG.

a

| 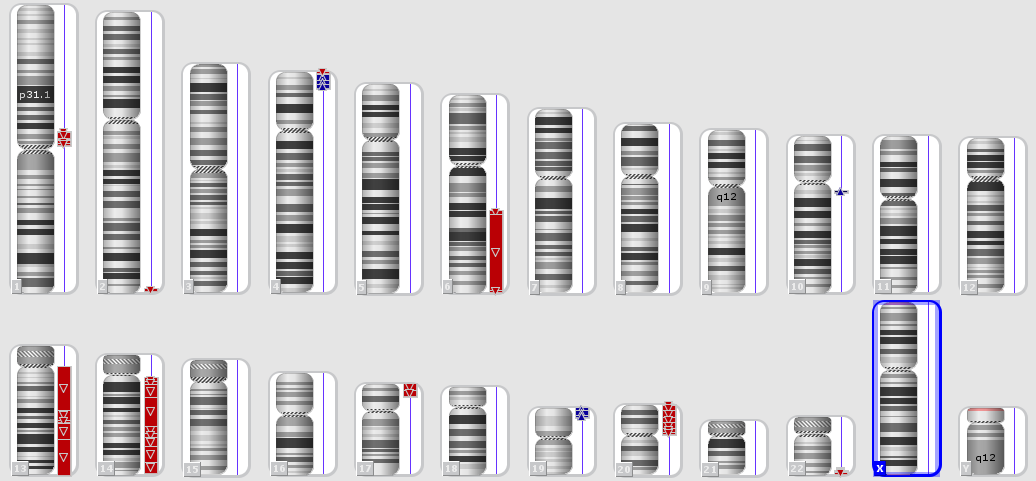  M#06_D | 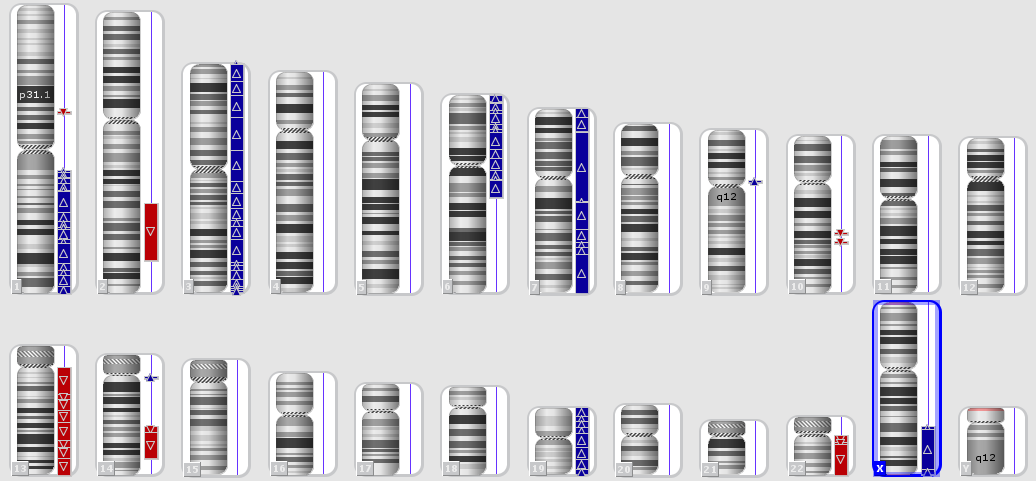  M#05_D  M#12_D | 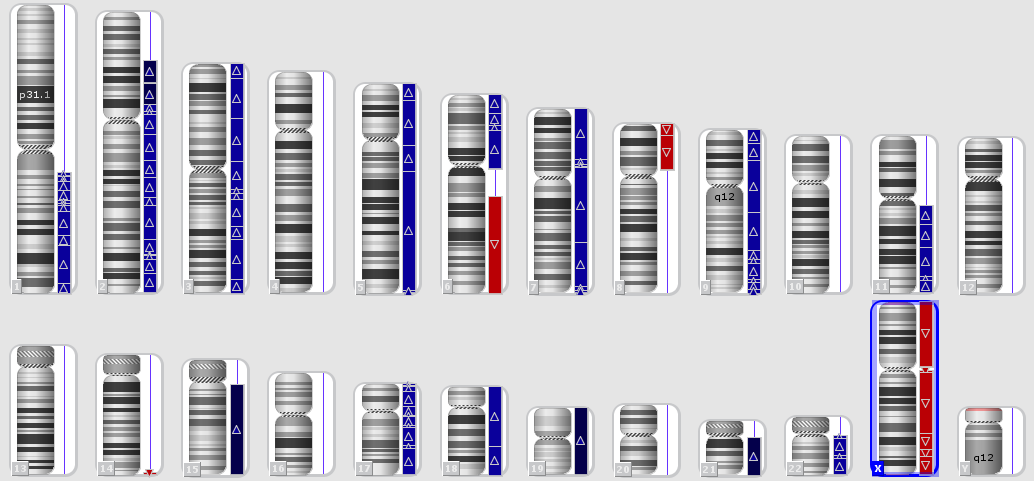  M#11_D  M#17_D  M#18_D |
| --- | --- | --- |
| 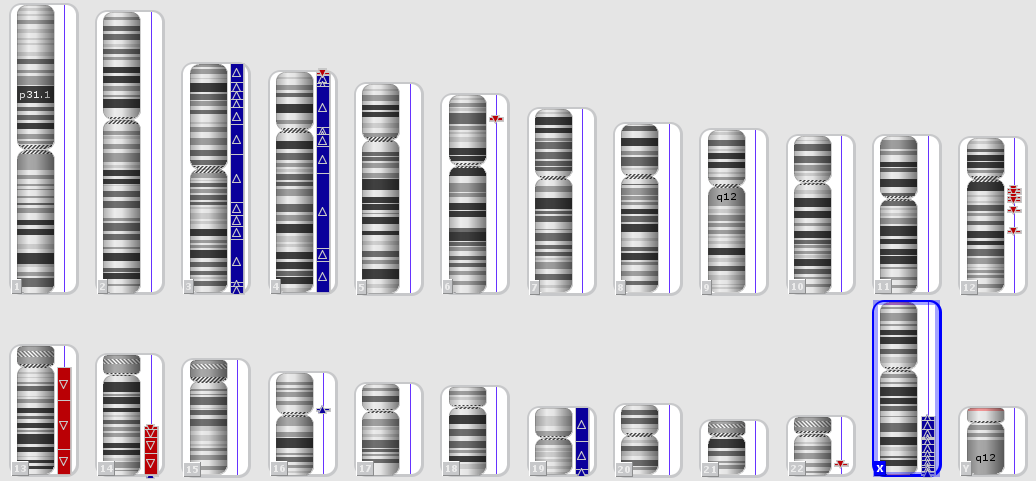 | 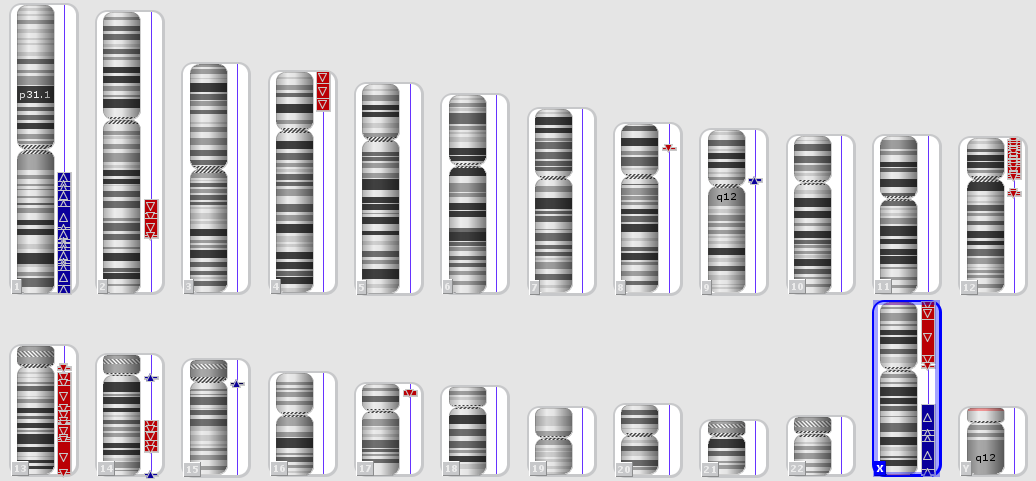 | 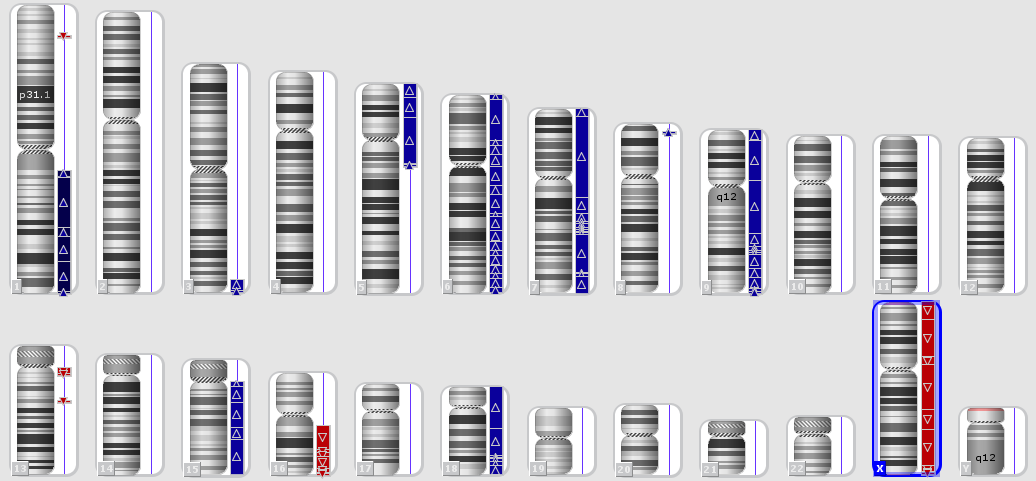 |
| 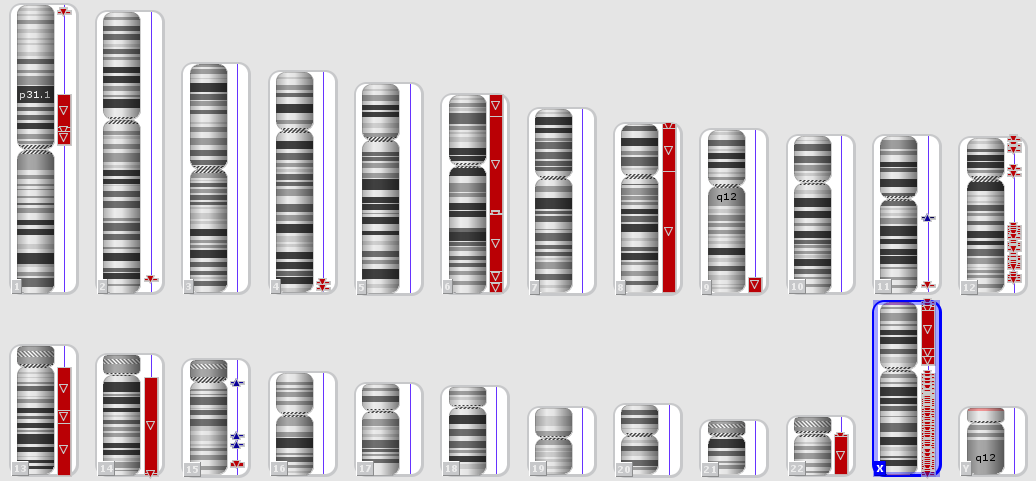  M#07_D | 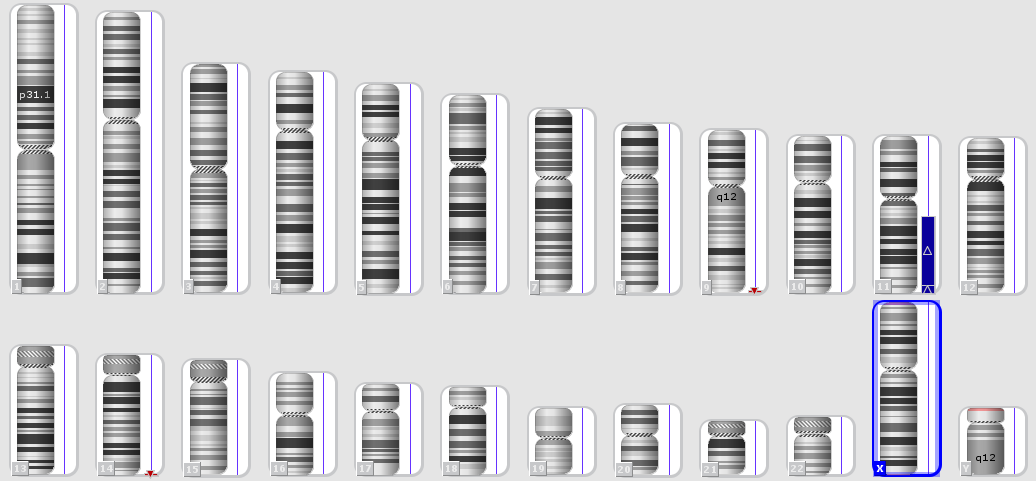  M#13_D  M#14_D | 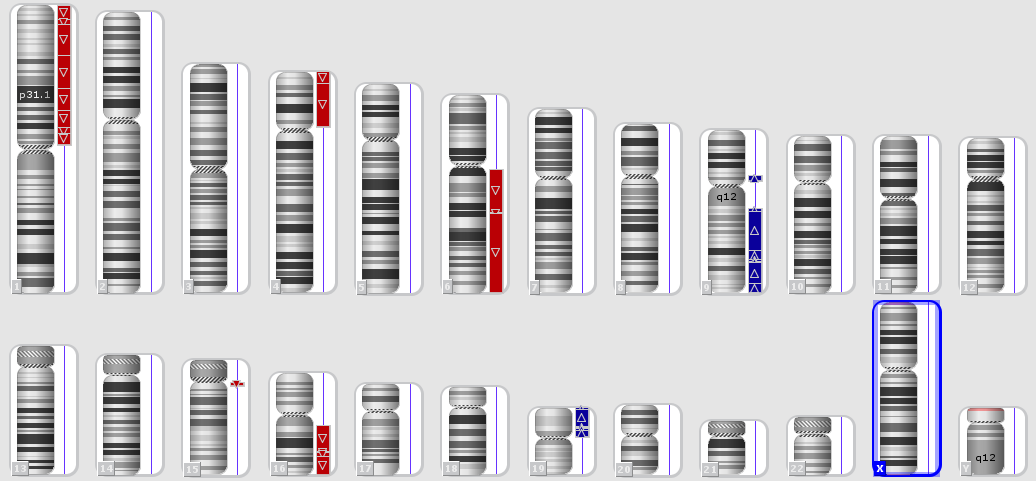  M#19_D  M#20_D |
| 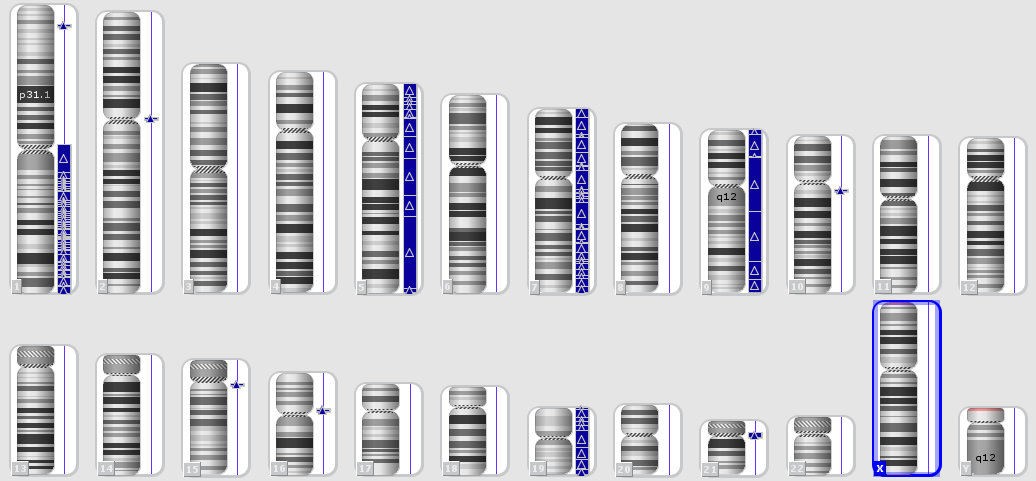  M#08_D | 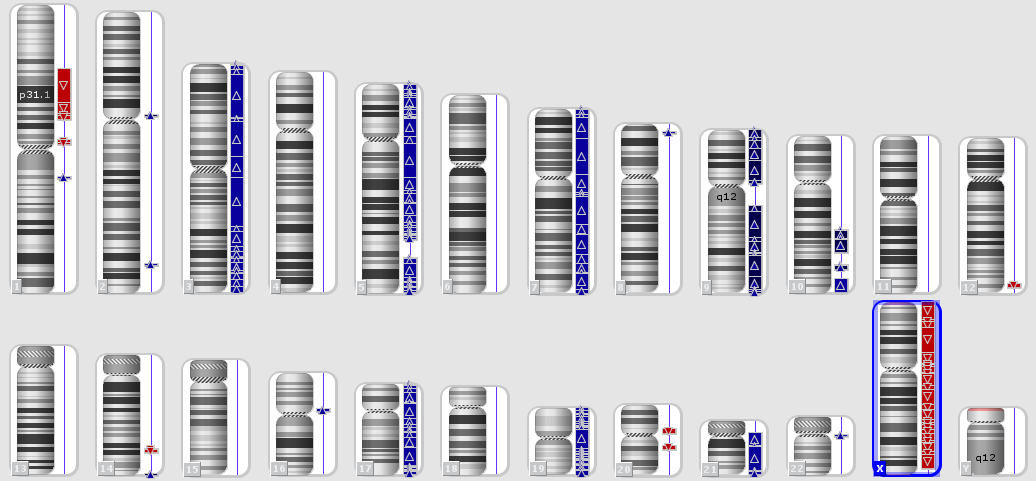 | 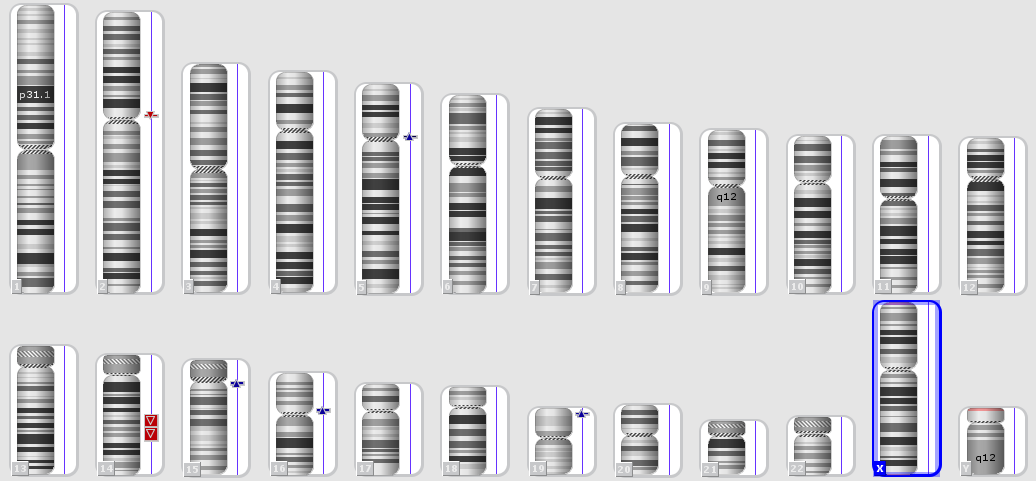 |
| 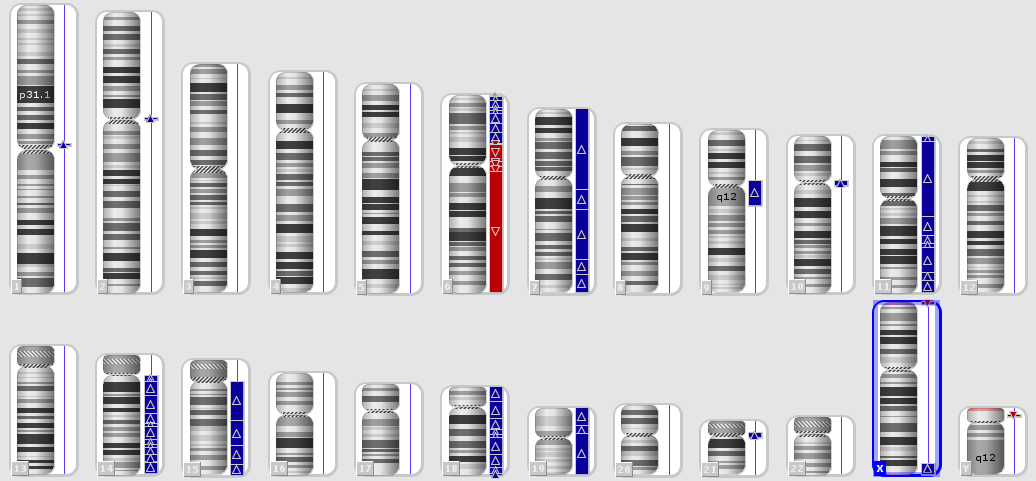  M#09_D | 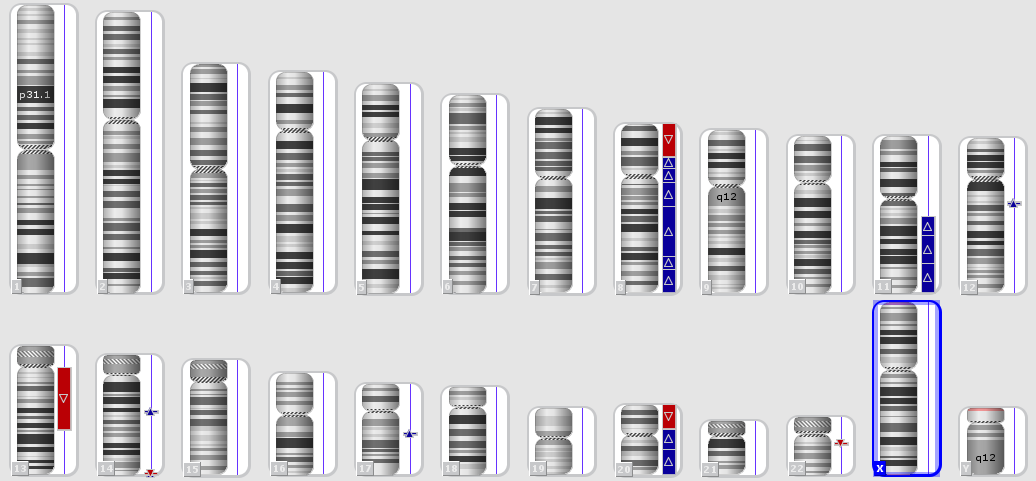  M#15_D  M#16_D | 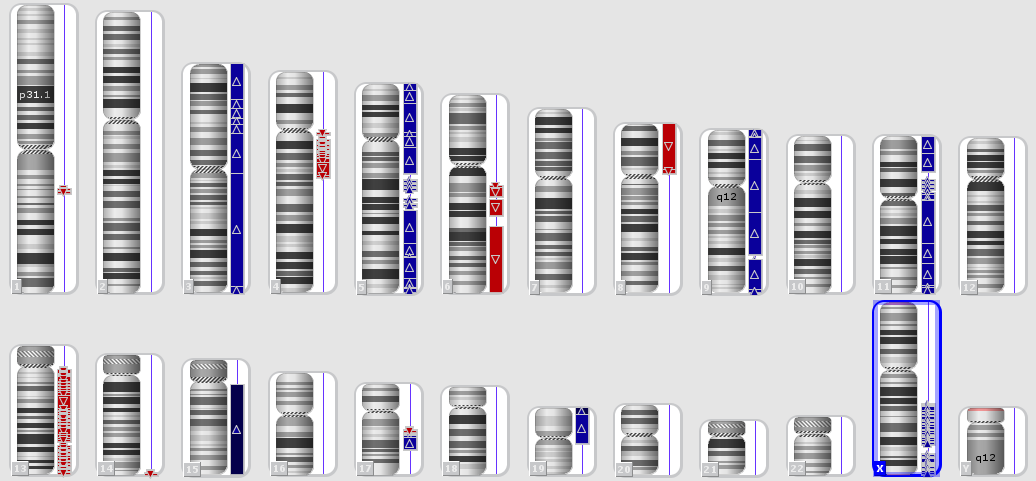  M#21_D |
| 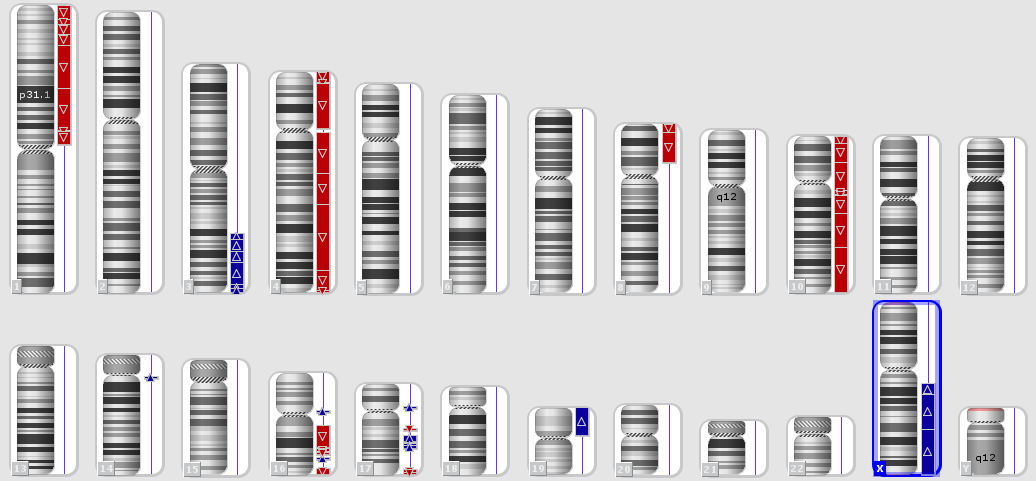  M#10_D | 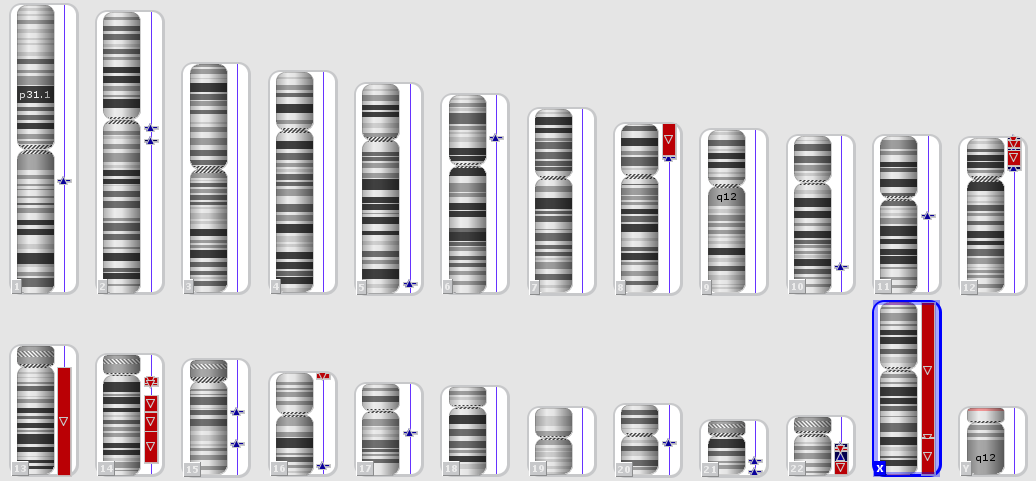 |  |

b


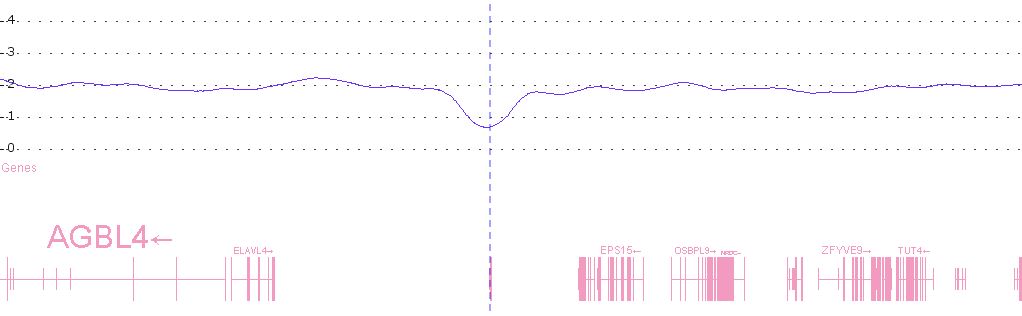


*CDKN2C*

**Figure S13 | Karyoview of the 17 diagnosed myeloma patients**. (a) Copy number analysis was performed to identify the copy number state of each patient. The karyoview presents a diagram of all CNVs (red bar for loss; blue bar for gain) of each patient. (b) M#11 : Copy number Smooth Signal of *CDKN2C* genomic locus .


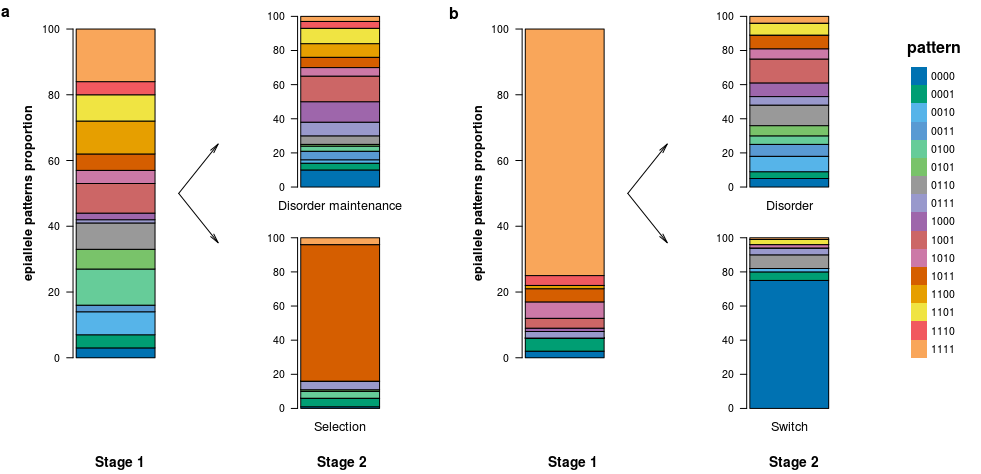


**Figure S14 |** Illustration of the four extreme changes in epiallele patterns between two stages: (a) Stage 1, heterogeneous epialleles; stage 2 (top), a similar heterogeneous pattern; stage 2 (bottom), selection of one pattern. (b) Stage 1, major fully methylated pattern; stage 2 (top), heterogeneous pattern; stage 2 (bottom), major fully unmethylated pattern. Legend for methylation patterns: 1 = methylated CpG and 0 = unmethylated CpG.


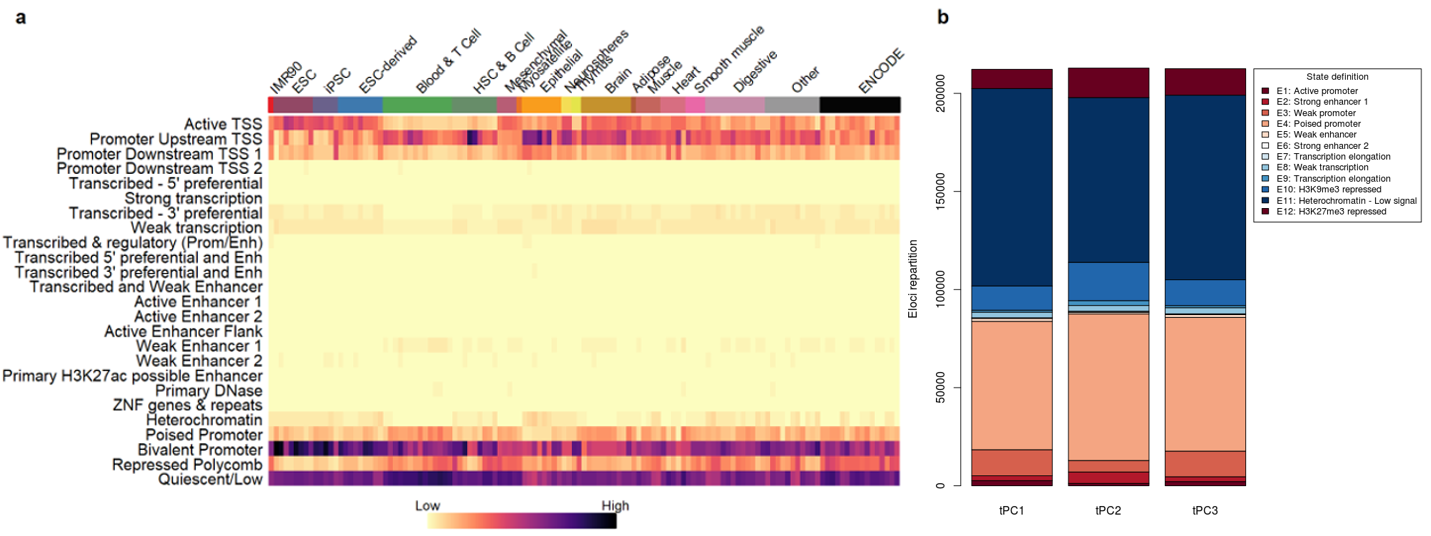


**Figure S15** | Eloci (NPC vs diagnosis) annotation according to the 25 chromatin states defined by ChromHMM for 127 epigenomes available on UCSC (a) and according to the 12 chromatin states defined by ChromHMM for plasma cell from tonsil (tPC) [58] (b).

**
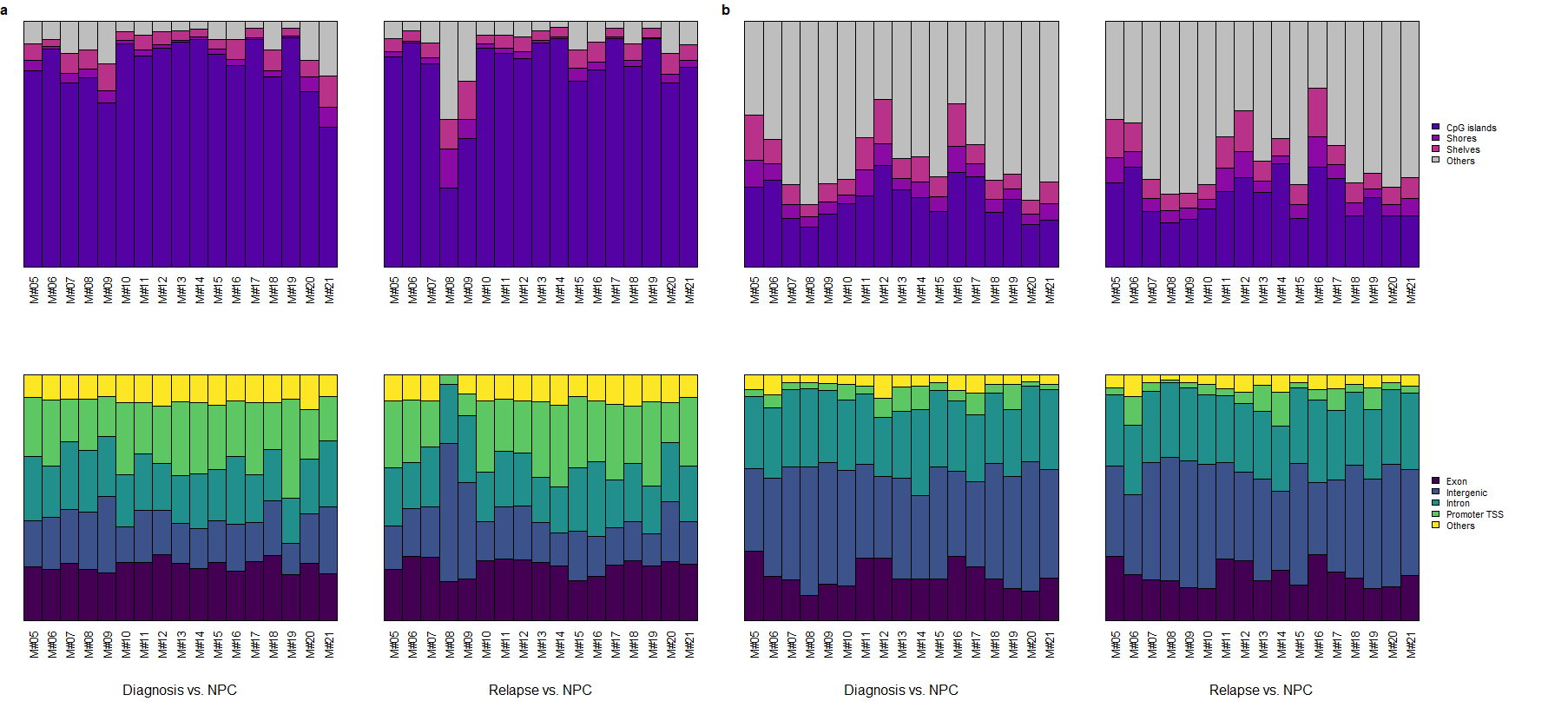
**

**Figure S16 |** Genomic distribution of NPCs versus diagnosis eloci and NPCs versus relapse eloci, in bivalent promoters (a) and quiescent regions (b), located in CGIs and adjacent regions (at the top) and in the main genomic regions: promoter TSS, exons, introns and intergenic regions (at the bottom).


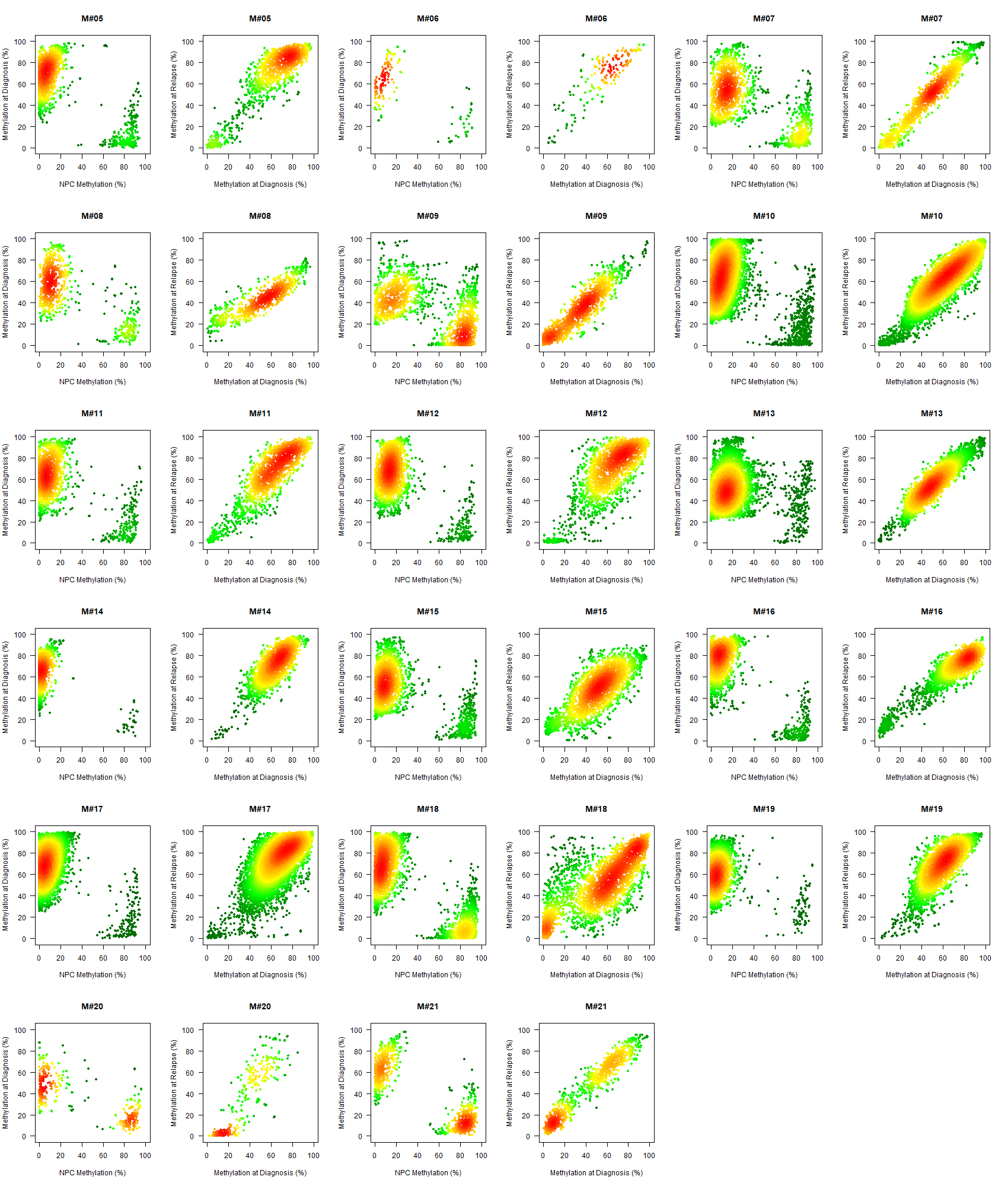


**Figure S17 |** Methylation disruption in bivalent promoters in MM. Scatterplot of eloci in bivalent promoters as a function of DNA methylation in NPCs and diagnosis samples (left figure) and as a function of DNA methylation at diagnosis and relapse (right figure) for all patients. The color gradient corresponds to the point density (low is green; high is red).

**
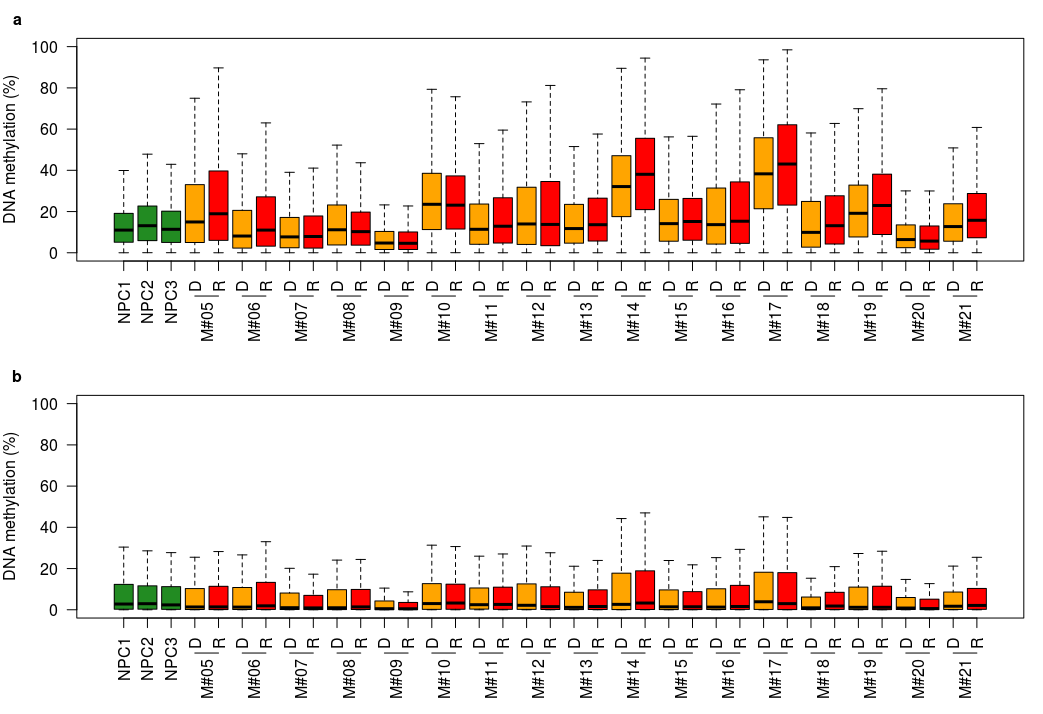
**

**Figure S18 |** Mean DNA methylation for bivalent promoter CGIs containing at least one hypermethylated elocus (a) or no elocus (b).


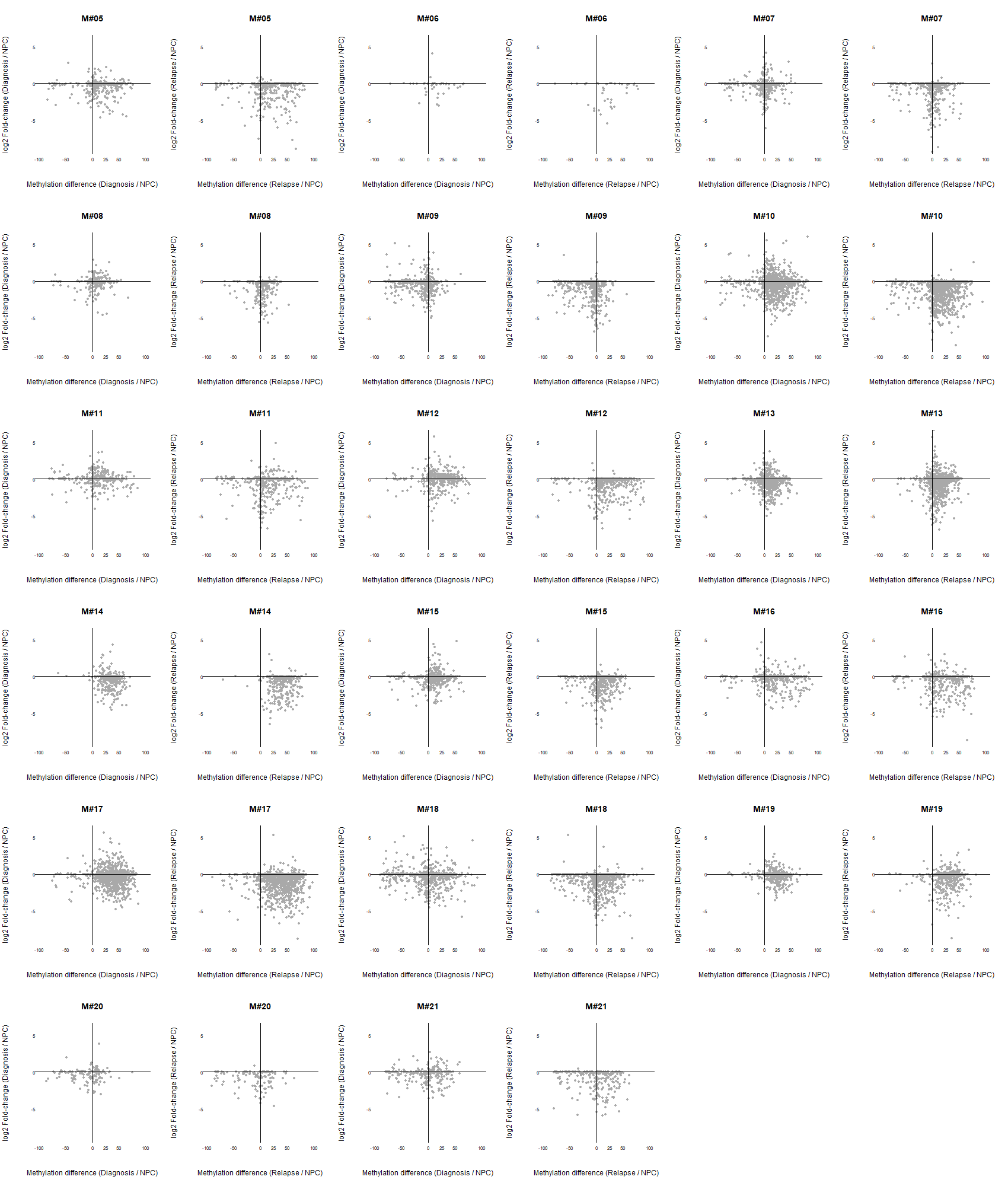


**Figure S19 |** Expression evolution of genes with eloci in bivalent promoters. Scatterplot of genes depending on the methylation level in the promoter and expression levels (left: diagnosis/NPC; right: relapse/NPC).


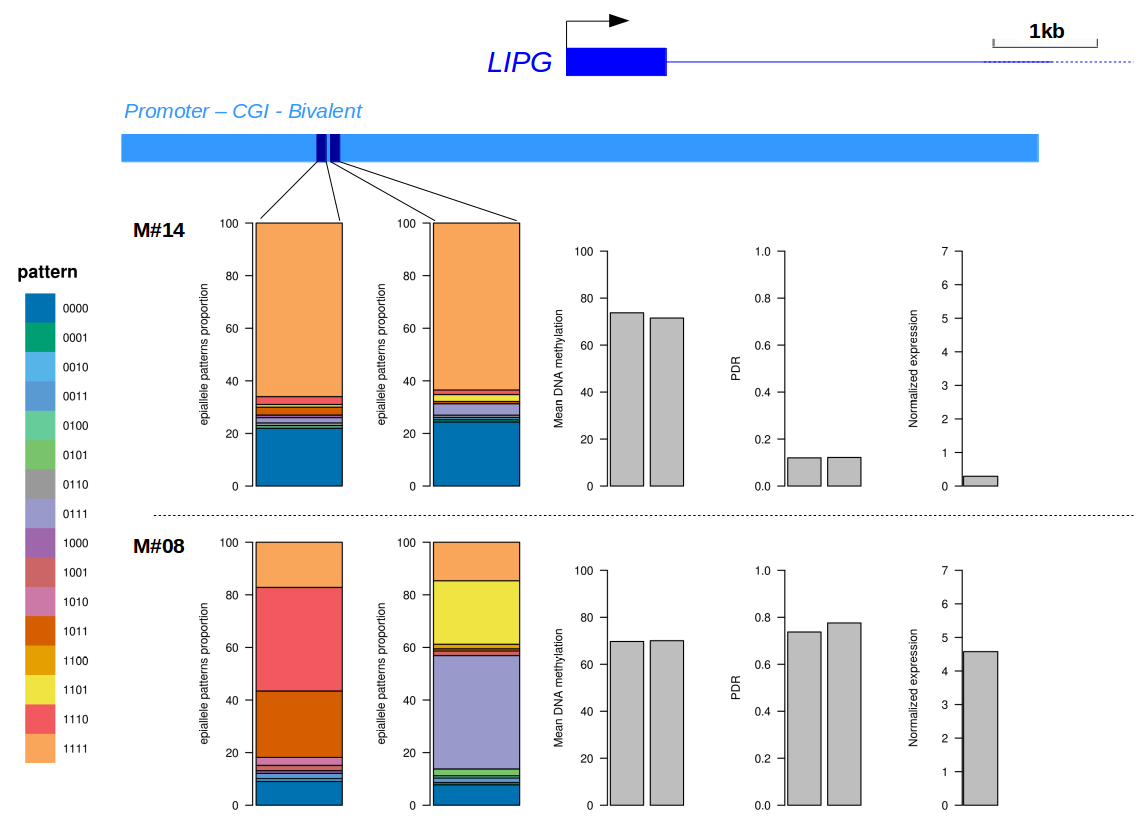


**Figure S20 |** Example of the decoupling relationship between promoter methylation and gene expression. The promoter region of *LIPG* shows comparable methylation levels in two samples (M#14 and M#08) but different PDR and expression levels (right). Epiallele patterns of two loci are shown in the illustration (left). Legend for methylation patterns: 1 = methylated CpG and 0 = unmethylated CpG.


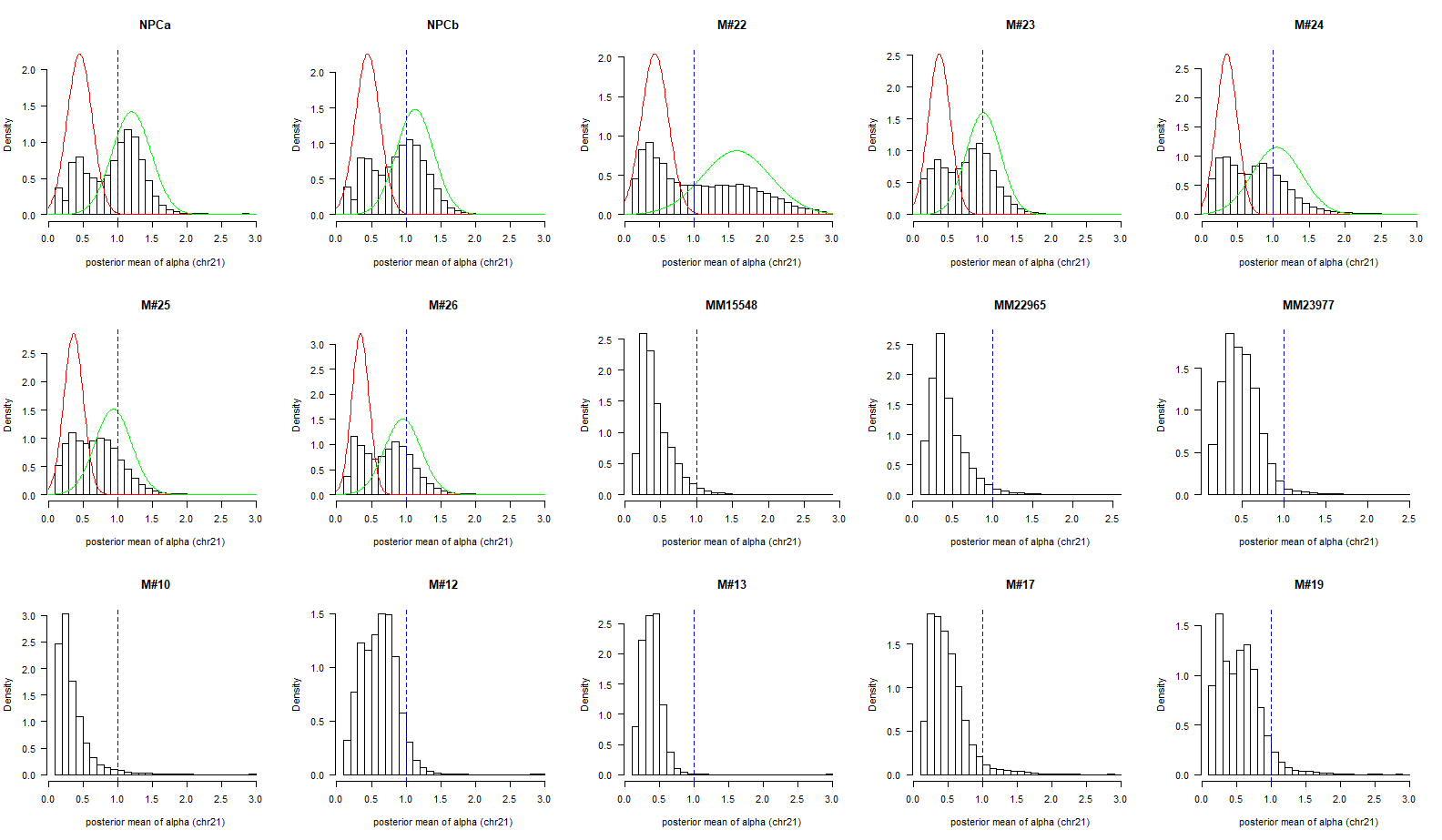


**Figure S21 |** Distribution of the α-value for chromosome 21. This distribution is similar for all chromosomes. The α-value characterizes the distribution of methylation levels in genomic windows of 100 CpGs. If the distribution of α-values is bimodal or has a large fraction of α value greater than or equal to 1 (blue dotted line), then this is evidence of the presence of PMDs. In the presence of a bimodal distribution, it is possible to calculate the PMDs on the genome with a hidden Markov model, whose two adjusted Gaussian distributions are represented in green and red.


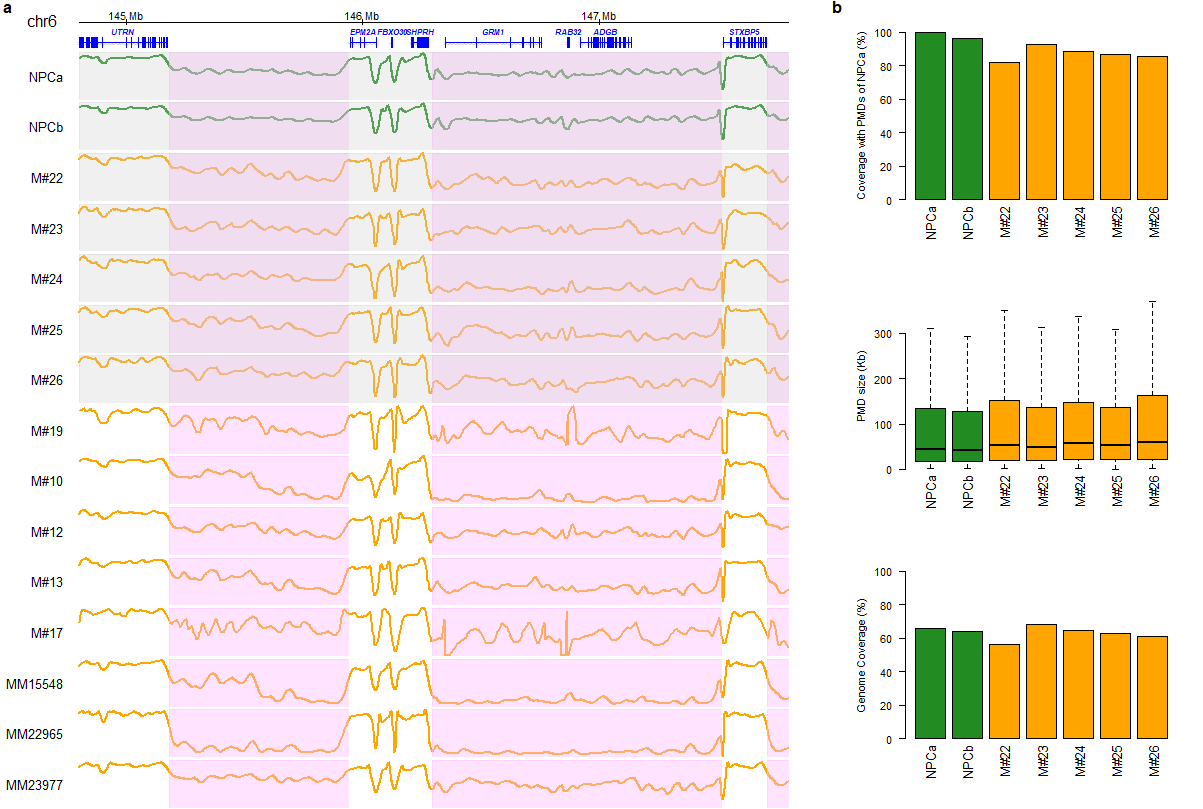


**Figure S22 |** Comparison of NPC and MM PMDs. (a) WGBS DNA methylation visualization in a genomic region encompassing two PMDs (pink area) in sample NPCa. Green lanes correspond to NPC methylation profiles, and orange lanes correspond to MM methylation profiles at diagnosis. The shaded profiles correspond to samples in which PMDs could be detected. (b) General characteristics of PMDs.


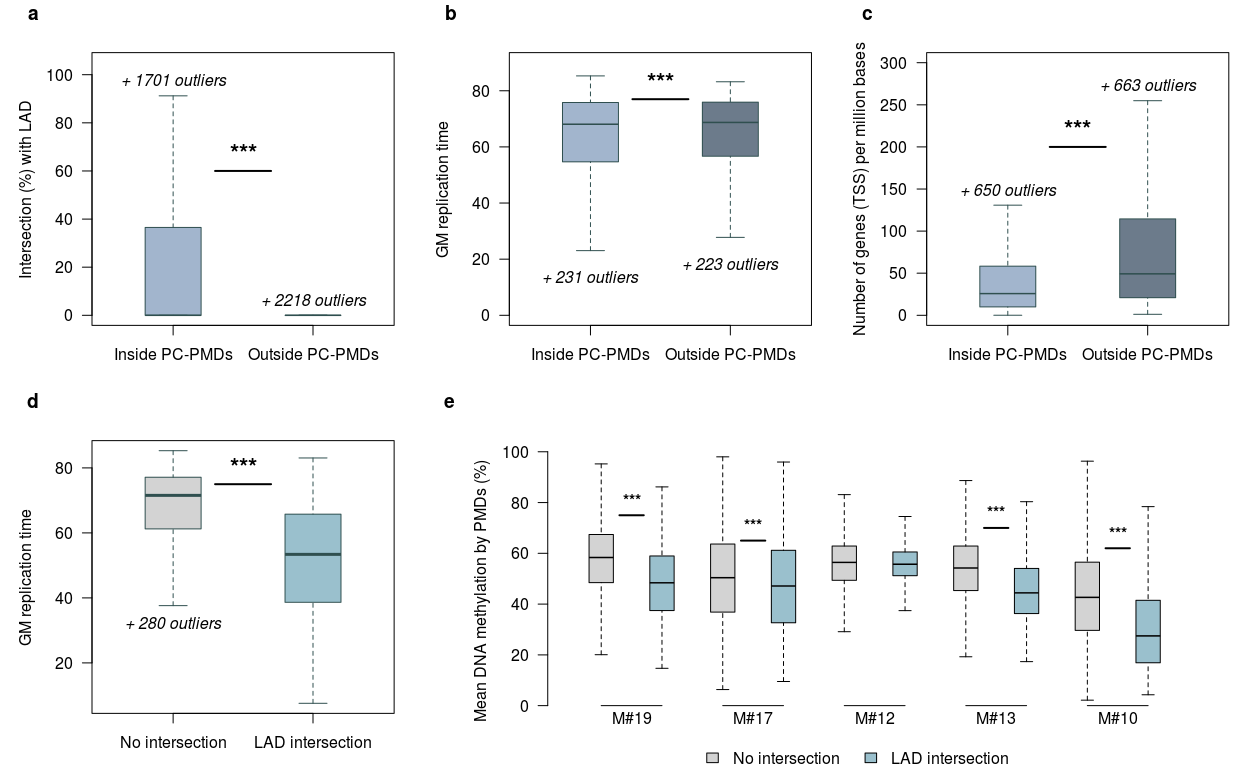


**Figure S23 |** Characteristics of PC-PMDs. (a) Percentage of intersections between LADs [68] and regions inside and outside PC-PMDs. (b) Replication timing of the GM12878 cell line (ENCODE data) inside and outside PC-PMDs. (c) Number of genes per million bases inside and outside PC-PMDs. (d) Replication timing of the GM12878 cell line in PC-PMDs intersecting (or not) with an LAD. (e) Mean DNA methylation level in PC-PMDs intersecting (or not) with an LAD per patient.


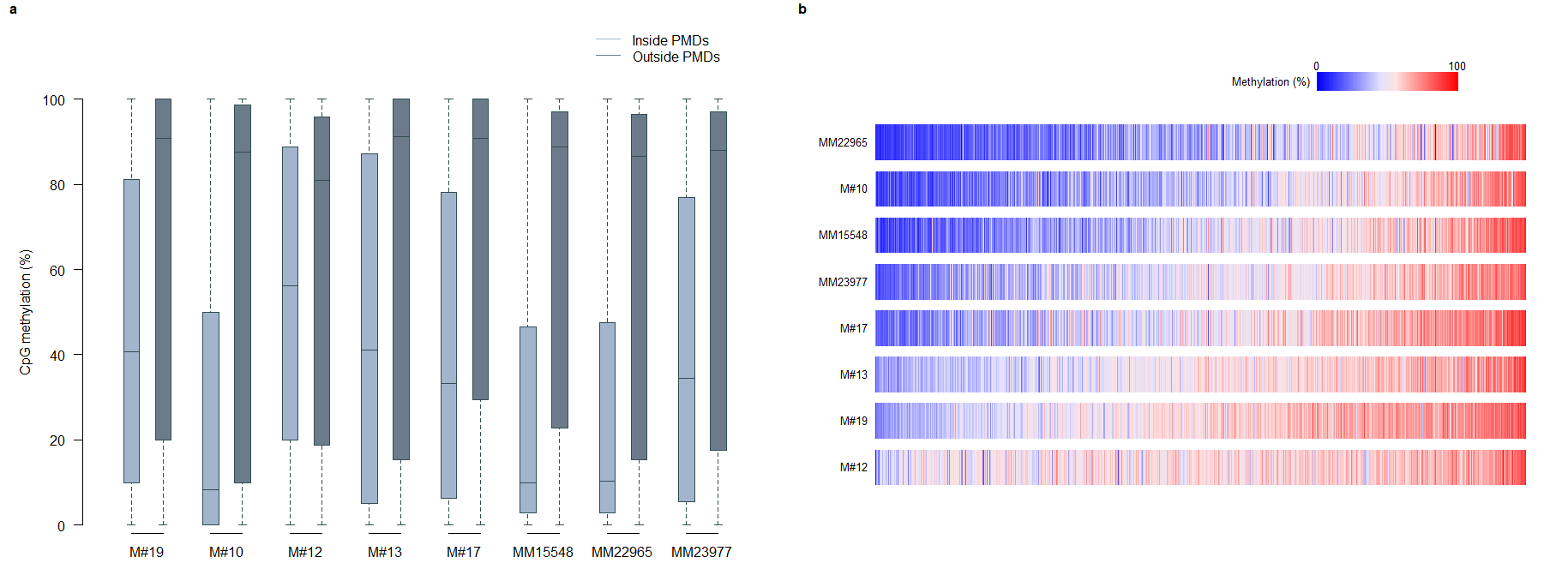


**Figure S24 |** Interpatient variability of PC-PMDs methylation. (a) DNA methylation levels of WGBS samples inside PC-PMD regions and outside PC-PMD regions (data from the BLUEPRINT project, see Additional file 1: Table S1). (b) Representation of the global DNA methylation of PC-PMDs in WGBS samples.

**
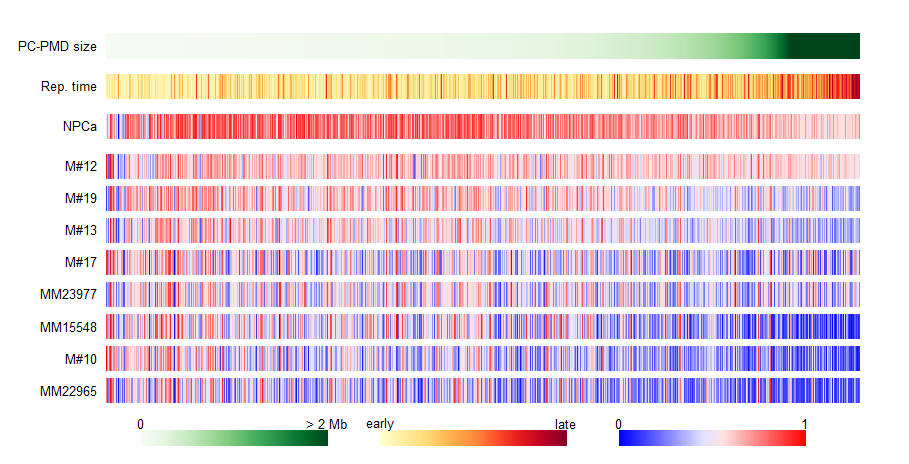
**

**Figure S25 |** Heatmap of PC-PMDs, sorted by increasing size, associated with GM12878 replication timing (ENCODE data) and DNA methylation levels in WGBS samples (NPC and diagnosis).


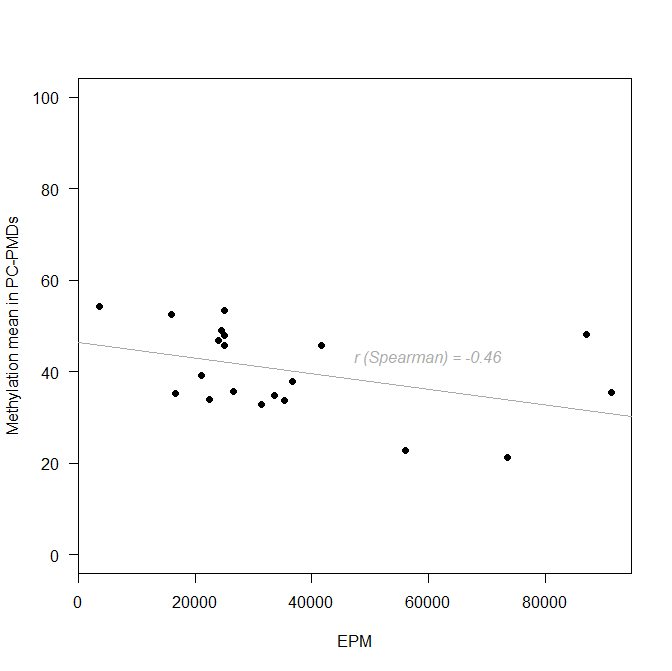


**Figure S26 |** Correlation between EPM and DNA methylation in PC-PMDs.


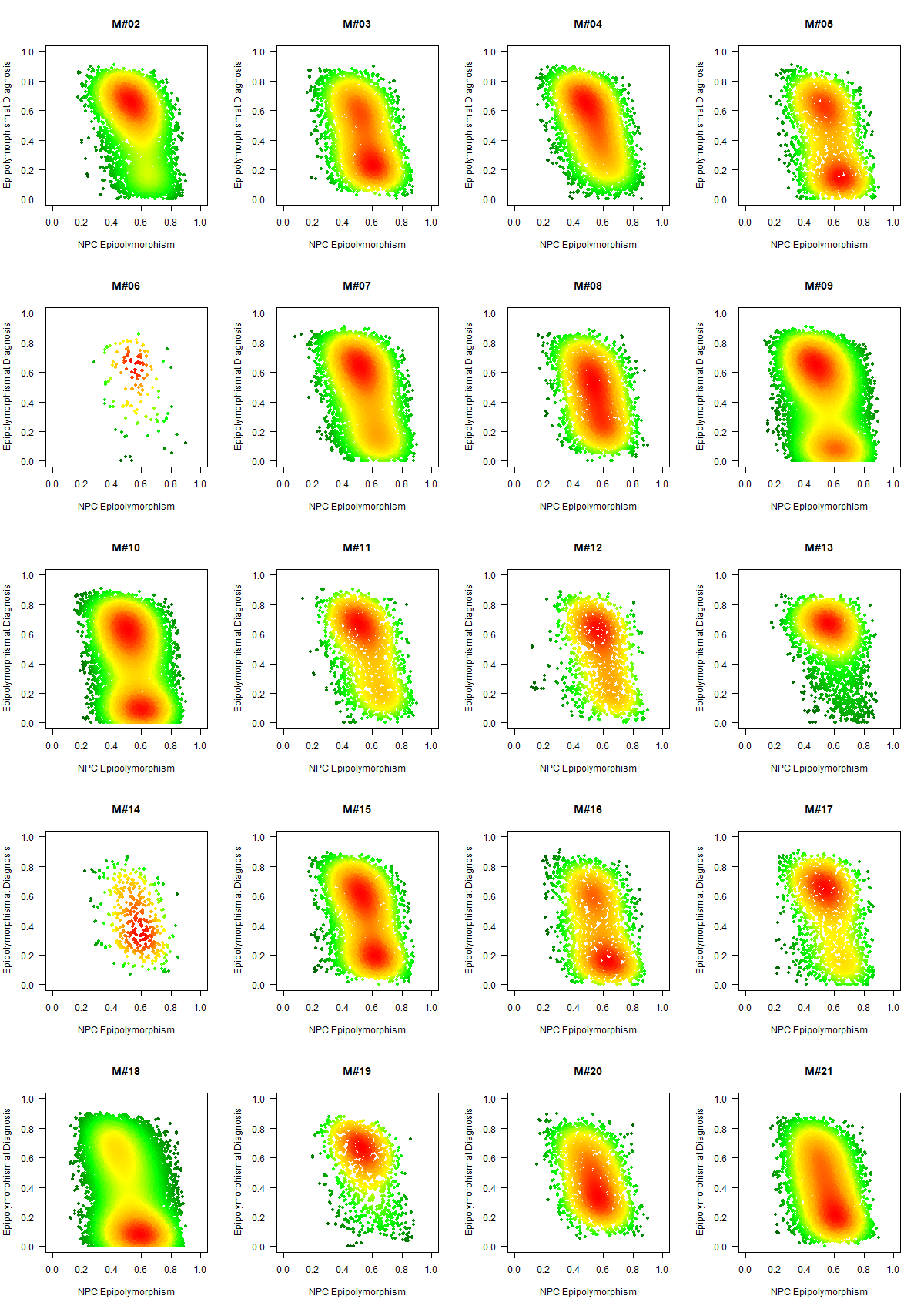


**Figure S27 |** Characterization of hypomethylated eloci epipolymorphism evolution. Scatterplot of eloci as a function of their epipolymorphism in NPC and diagnosis samples for all patients. The color gradient corresponds to the point density (low is green; high is red).

**
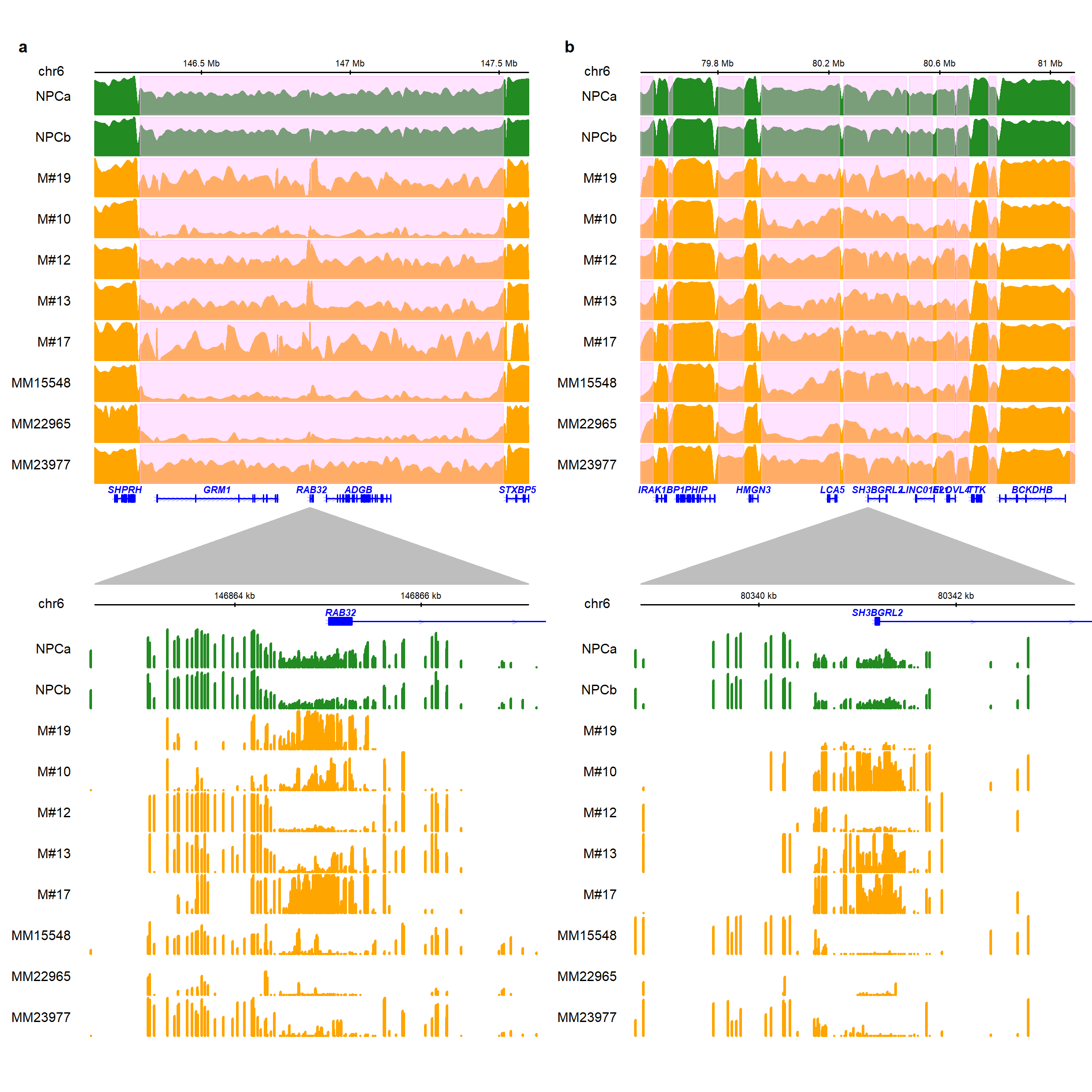
**

**Figure S28 |** Examples of genes with bivalent promoter CGIs with disrupted DNA methylation regions embedded within large partially methylated regions (pink area): *RAB32* (a) and *SH3BGRL2* (b).


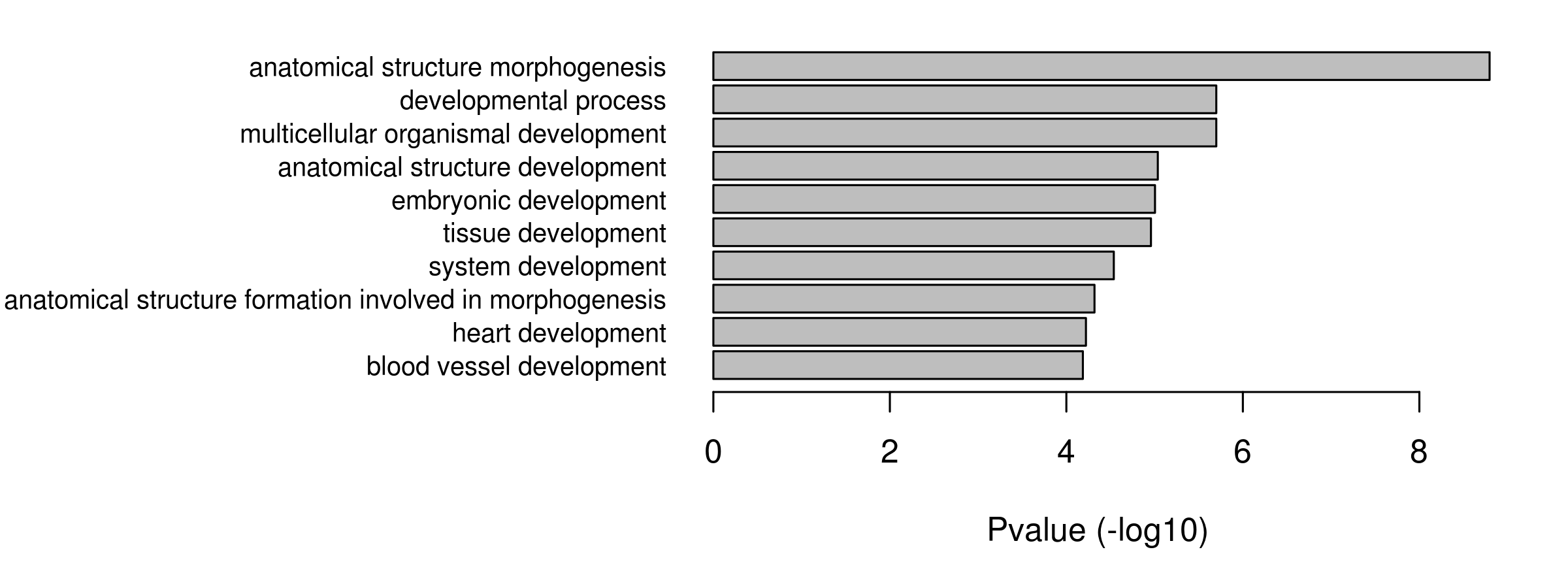


**Figure S29 |** Gene set enrichment analysis of genes from the list in the supplementary Table VII from Vicente-Dueñas et al [80].


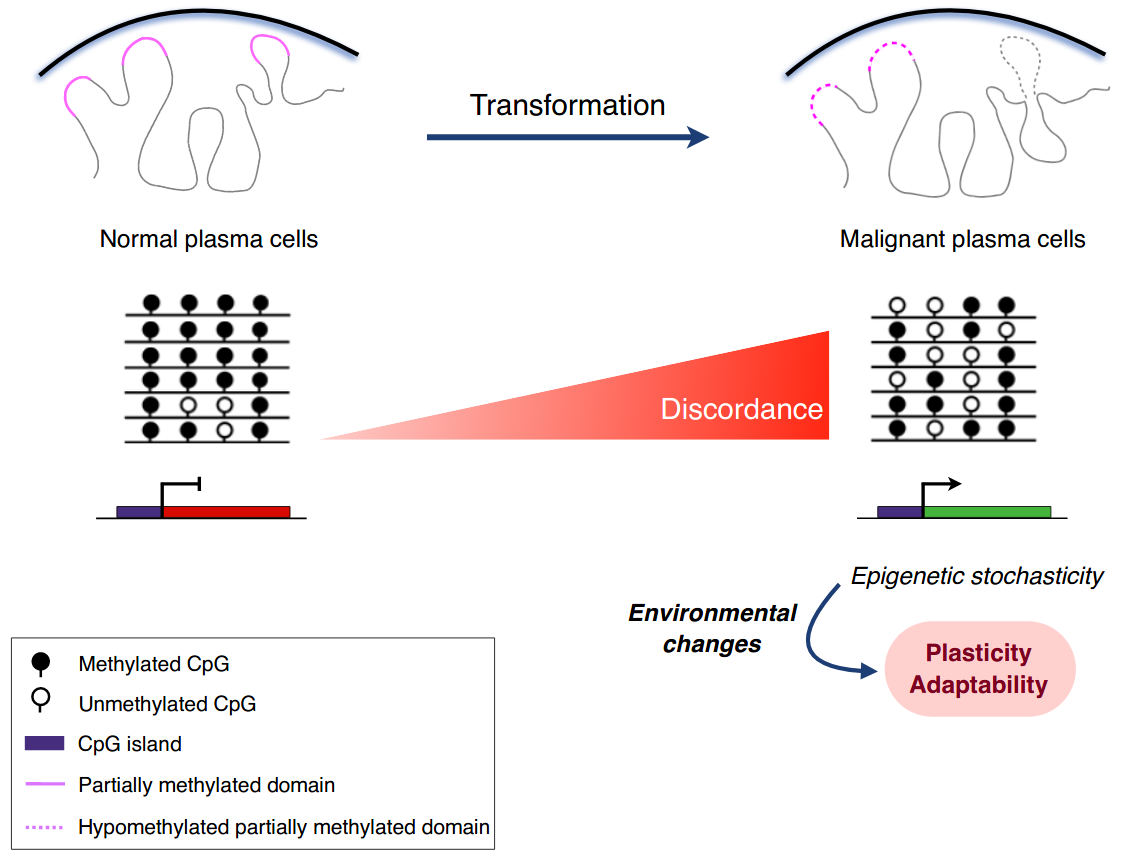


**Figure S30 |** A proposed model of the role of PMD instability in MM onset and development.

Malignant transformation is accompanied by increased hypomethylation of PMDs which contributes to epigenetic, transcriptomic and 3D organization heterogeneity across patients. The lack of accurate global DNA methylation maintenance also drives intrapatient DNA methylation heterogeneity which contributes to a decoupling relationship between promoter methylation and gene expression (genes with methylated promoter but not repressed) and transcriptomic variability. We therefore hypothesize that this epigenetic stochasticity could provide a selective advantage to the tumor cell by increasing its plasticity and adaptation to environmental changes.
